# Supplementary material for: Potentially avoidable causes of hospitalisation in people with dementia: contemporaneous associations by stage of dementia in a South London clinical cohort
Source: BMJ Open. 2022 Apr 4;12(4):e055447. doi: 10.1136/bmjopen-2021-055447 (PMC8984034; doi:10.1136/bmjopen-2021-055447)
Supplement: Supplementary data [file bmjopen-2021-055447supp001.pdf]

Supplementary Table 1: Ambulatory care sensitive conditions*

ACSC	ICD code position		ICD-10 codes
	Main analysis	Sensitivity analysis	
**Angina**	Principal	1° or 2°	I20, I200, I201, I208, I209, I240, I248, I249, I25, I250, I251, I252, I253, I254, I255, I256, I258, I259
**Asthma**	Principal	1° or 2°	J45, J450, J451, J458, J459, J46, J46X
**Cellulitis**	Principal	1° or 2°	L03, L030, L031, L032, L033, L038, L039, L04, L040, L041, L042, L043, L048, L049, L08, L080, L081, L088, L089, L88, L88X, L980, L983
**Congestive heart failure**	Principal	1° or 2°	I110, I13, I130, I131, I132, I139, I50, I500, I501, I509, J81, J81X
**Convulsions and epilepsy**	Principal	1° or 2°	G40, G400, G401, G402, G403, G404, G405, G406, G407, G408, G409, G41, G410, G411, G412, G418, G419, O15, R56, R560, R568
**COPD**	Principal	1° or 2°	J20, J41, J410, J411, J418, J42, J42X, J43, J430, J431, J432, J438, J439, J44, J440, J441, J448, J449, J47, J47X
**Dehydration**	Principal	1° or 2°	E86, E86X
**Dental conditions**	Principal	1° or 2°	A690, K02, K020, K021, K022, K023, K024, K028, K029, K03, K030, K031, K032, K033, K034, K035, K036, K037, K038, K039, K04, K040, K041, K042, K043, K044, K045, K046, K047, K048, K049, K05, K050, K051, K052, K053, K054, K055, K056, K06, K060, K061, K062, K068, K069, K08, K080, K081, K082, K083, K088, K089, K09, K090, K091, K092, K098, K099, K12, K120, K121, K122, K13, K130, K131, K132, K133, K134, K135, K136, K137
**Diabetes complications**	Any	Any	E10, E100, E101, E102, E103, E104, E105, E106, E107, E108, E11, E110, E111, E112, E113, E114, E115, E116, E117, E118, E12, E120, E121, E122, E123, E124, E125, E126, E127, E128, E13, E130, E131, E132, E133, E134, E135, E136, E137, E138, E14, E140, E141, E142, E143, E144, E145, E146, E147, E148
**Ear, nose and throat infections**	Principal	1° or 2°	H66, H660, H661, H662, H663, H664, H669, H67, H670, H671, H678, J02, J020, J028, J029, J03, J030, J038, J039, J06, J060, J068, J069, J312
**Gangrene**	Any	Any	R02
**Gastroenteritis**	Principal	1° or 2°	K522, K528, K529
**Hypertension**	Principal	1° or 2°	I10, I10X, I119, I129, I139
**Influenza**	Any	Any	J10, J100, J101, J108, J11, J110, J111, J118
**Iron deficiency anaemia**	Principal	1° or 2°	D50, D501, D508, D509
**Nutritional deficiencies**	Principal	1° or 2°	E40, E40X, E41, E41X, E42, E42X, E43, E43X, E55, E550, E559, E64, E640, E641, E642, E643, E648, E649
**Nutritional deficiencies OR other nutritional deficiency anaemia**	Principal	1° or 2°	D51, D510, D511, D512, D513, D518, D519
**Other vaccine preventable**	Any	Any	A35, A35X, A36, A360, A361, A362, A363, A368, A369, A37, A370, A390, A80, A801, A802, A803, A804, A809, B05, B050, B051, B052, B053, B054, B058, B059, B06, B060, B068, B069, B16, B160, B161, B162, B169, B180, B181, B26, B260, B261, B262, B263, B268, B269, G000, G001, M010, M014
**Pelvic inflammatory disease**	Principal	1° or 2°	N70, N700, N701, N709, N73, N730, N731, N732, N733, N734, N735, N736, N738, N739, N74, N740, N741, N742, N743, N744, N748
**Perforated/bleedi ng ulcer**	Principal	1° or 2°	K25, K250, K251, K252, K254, K255, K256, K26, K260, K261, K262, K264, K265, K266, K27, K270, K271, K272, K274, K275, K276, K28, K280, K281, K282, K284, K286
**Pneumonia**	Any	Any	J13, J14, J153, J154, J157, J159, J168, J18, J180, J181, J182, J188, J189
**Tuberculosis**	Principal	1° or 2°	A15, A150, A151, A152, A153, A154, A155, A156, A157, A158, A159, A16, A160, A161, A162, A163, A164, A165, A167, A168, A169, A19, A190, A191, A192, A198, A199
**Urinary tract infection /Pyelonephritis**	Principal	1° or 2°	N10, N10X, N11, N110, N111, N118, N119, N12, N12X, N136, N30, N300, N301, N302, N303, N304, N308, N309, N390

ACSC: ambulatory care sensitive condition

*This table was created by the authors, however, the ACSCs, the respective ICD-10 codes and their position were initially based on those published by Bardsley *et al*, 2013. Codes were augmented with those published by the UK Health and Social Care Information Centre and by the authors. Deviations from the approach of Bardsley *et al* comprised of: i) cystitis codes were added to the UTI category; ii) O15 (eclampsia) was removed from the convulsions and epilepsy category. The approach used in the sensitivity was determined by the authors.

Supplementary Table 2: Results from stepwise-selected generalised ordinal logistic regression model for severity of dementia (sensitivity analysis *)

	Moderate/severe vs. mild dementia		Severe vs. mild/moderate dementia	
	**OR (95% CI)**	**p**	**OR (95% CI)**	**p**
**Sex (male reference)**	1.09 (0.97, 1.21)	0.141	1.09 (0.97, 1.21)	0.141
**Time since first presentation at SLaM**	1.13 (1.08, 1.18)	<0.001	1.13 (1.08, 1.18)	<0.001
**Age group at index date**	1.21 (1.11, 1.31)	<0.001	0.99 (0.89, 1.11)	0.856
**Ethnic group (European reference)**				
	1.21 (1.06, 1.38)	0.005	1.21 (1.06, 1.38)	0.005
**Dementia diagnostic group (AD reference)**				
Vascular	1.33 (1.15, 1.56)	<0.001	1.02 (0.85, 1.24)	0.816
Mixed	1.15 (0.95, 1.4)	0.163	0.8 (0.61, 1.05)	0.107
Other	1.11 (0.98, 1.27)	0.112	1.11 (0.98, 1.27)	0.112
**Deprivation score**	1.12 (1.06, 1.17)	<0.001	1.12 (1.06, 1.17)	<0.001
**Convulsions**	1.58 (0.96, 2.58)	0.07	1.58 (0.96, 2.58)	0.07
**Iron deficiency anaemia**	1.23 (0.63, 2.4)	0.542	0.25 (0.06, 1.05)	0.058
**Perforated/bleeding ulcer**	6.92 (1.56, 30.77)	0.011	6.92 (1.56, 30.77)	0.011
**Pneumonia**	1.38 (1.12, 1.69)	0.002	1.38 (1.12, 1.69)	0.002
**UTI pyelonephritis**	1.29 (1.1, 1.51)	0.002	1.29 (1.1, 1.51)	0.002

Goodness of fit: LR Chi square 143.3, $p<0.0001$
ACSC: ambulatory care sensitive condition
*Criteria were relaxed to include diagnoses in either of the first two positions for ACSCs that were initially defined based on a diagnosis in the primary position only.
