# Supplementary material for: Potentially avoidable causes of hospitalisation in people with dementia: contemporaneous associations by stage of dementia in a South London clinical cohort
Source: BMJ Open. 2022 Apr 4;12(4):e055447. doi: 10.1136/bmjopen-2021-055447 (PMC8984034; doi:10.1136/bmjopen-2021-055447)
Supplement: Author's manuscript [file bmjopen-2021-055447.draft_revisions.pdf]

BMJ Open is committed to open peer review. As part of this commitment we make the peer review history of every article we publish publicly available.

When an article is published we post the peer reviewers' comments and the authors' responses online. We also post the versions of the paper that were used during peer review. These are the versions that the peer review comments apply to.

The versions of the paper that follow are the versions that were submitted during the peer review process. They are not the versions of record or the final published versions. They should not be cited or distributed as the published version of this manuscript.

BMJ Open is an open access journal and the full, final, typeset and author-corrected version of record of the manuscript is available on our site with no access controls, subscription charges or pay-per-view fees (http://bmjopen.bmj.com).

If you have any questions on BMJ Open's open peer review process please email info.bmjopen@bmj.com

BMJ Open

POTENTIALLY AVOIDABLE CAUSES OF HOSPITALISATION IN A CLINICAL COHORT OF PATIENTS WITH MILD, MODERATE AND SEVERE DEMENTIA

Journal:	*BMJ Open*
Manuscript ID	bmjopen-2021-055447
Article Type:	Original research
Date Submitted by the Author:	14-Jul-2021
Complete List of Authors:	Gungabissoon, Usha; GSK, Epidemiology (Value Evidence and Outcomes); King's College London Institute of Psychiatry Psychology and Neuroscience, Psychological Medicine<br>Perera, Gayan; King's College London Institute of Psychiatry Psychology and Neuroscience, Psychological Medicine<br>Galwey, Nicholas; GSK, Research Statistics<br>Stewart, Robert; King's College London Institute of Psychiatry Psychology and Neuroscience, Psychological Medicine; South London and Maudsley NHS Foundation Trust
Keywords:	Dementia < NEUROLOGY, EPIDEMIOLOGY, Old age psychiatry < PSYCHIATRY

SCHOLARONE™
Manuscripts

I, the Submitting Author has the right to grant and does grant on behalf of all authors of the Work (as defined in the below author licence), an exclusive licence and/or a non-exclusive licence for contributions from authors who are: i) UK Crown employees; ii) where BMJ has agreed a CC-BY licence shall apply, and/or iii) in accordance with the terms applicable for US Federal Government officers or employees acting as part of their official duties; on a worldwide, perpetual, irrevocable, royalty-free basis to BMJ Publishing Group Ltd ("BMJ") its licensees and where the relevant Journal is co-owned by BMJ to the co-owners of the Journal, to publish the Work in this journal and any other BMJ products and to exploit all rights, as set out in our licence.

The Submitting Author accepts and understands that any supply made under these terms is made by BMJ to the Submitting Author unless you are acting as an employee on behalf of your employer or a postgraduate student of an affiliated institution which is paying any applicable article publishing charge ("APC") for Open Access articles. Where the Submitting Author wishes to make the Work available on an Open Access basis (and intends to pay the relevant APC), the terms of reuse of such Open Access shall be governed by a Creative Commons licence – details of these licences and which Creative Commons licence will apply to this Work are set out in our licence referred to above.

Other than as permitted in any relevant BMJ Author's Self Archiving Policies, I confirm this Work has not been accepted for publication elsewhere, is not being considered for publication elsewhere and does not duplicate material already published. I confirm all authors consent to publication of this Work and authorise the granting of this licence.

For peer review only

WORD COUNT: 3719

TITLE: POTENTIALLY AVOIDABLE CAUSES OF HOSPITALISATION IN A CLINICAL COHORT OF PATIENTS WITH MILD, MODERATE AND SEVERE DEMENTIA

Authors:

Usha Gungabissoon[1,2], M.Sc.; Gayan Perera[1], Ph.D.; Nicholas Galwey[3], Ph.D.; Robert Stewart[1,4], M.D., FRCPsych

[1]Institute of Psychiatry, Psychology and Neuroscience (IOPPN), King's College London, London, United Kingdom (UK)

[2]Epidemiology, Value, Evidence and Outcomes, Global Medical, GlaxoSmithKline (GSK) R&D, London, UK

[3]Research Statistics, GSK R&D, London, UK

[4]South London and Maudsley NHS Foundation Trust, London, UK

For peer review only

For peer review only - http://bmjopen.bmj.com/site/about/guidelines.xhtml

Corresponding author: Usha Gungabissoon

Department of Psychological Medicine (PO84), Institute of Psychiatry, Psychology and Neuroscience, King's College London. De Crespigny Park, London SE5 8AF

Email: usha.2.gungabissoon@gsk.com

Phone: +442080476878

For peer review only

For peer review only - http://bmjopen.bmj.com/site/about/guidelines.xhtml

ABSTRACT (word count 244/300)

Objectives

To estimate the frequency of all-cause hospitalisations and those related to ambulatory care sensitive conditions (ACSCs); a proxy for potentially avoidable hospitalisations amongst individuals with dementia, and to investigate differences by stage of dementia based on recorded cognitive function.

Setting

Data from a large London dementia care case register, linked to a national hospitalisation database.

Participants

Individuals aged ≥65 years with a confirmed dementia diagnosis with recorded cognitive function.

Outcome measures

Acute general hospital admissions were evaluated within 6 months of a randomly selected cognitive function score in patients with a clinical diagnosis of dementia. All-cause and ACSC-related hospital admissions were evaluated by stage of dementia.

Results

Of 5,294 people with dementia, 2,993 (56.5%) had at least one hospitalisation during a 12-month period of evaluation, and 1,192 (22.5%) had an ACSC-related admission. Proportions with an all-cause or ACSC-related hospitalisation were greater in the groups with more advanced dementia. An ACSC-related admission was associated with 1.3-fold increased odds of more severe dementia after adjusting for demographic factors. Concerning admissions for individual ACSCs, the most common ACSC was UTI/pyelonephritis (9.8% of hospitalised patients) followed by pneumonia (7.1%); in an adjusted model, these were each associated with 1.4-fold increased odds of more severe dementia.

Conclusions

For peer review only - http://bmjopen.bmj.com/site/about/guidelines.xhtml

Potentially avoidable hospitalisations were common in people with dementia, particularly in those with greater cognitive impairment. Our results call for greater attention to the extent of cognitive status impairment, and not just dementia diagnosis, when evaluating measures to prevent unnecessary hospitalisations.

Key words: dementia, epidemiology, healthcare utilisation, avoidable hospitalisation, ambulatory care sensitive

For peer review only

For peer review only - http://bmjopen.bmj.com/site/about/guidelines.xhtml

Strengths and limitations of this study

- We evaluated potentially avoidable causes of hospitalisation in a large sample of clinically confirmed cases of dementia in a catchment specialist healthcare case register linked to a national hospitalisation database providing near-complete outcome ascertainment. Severity of dementia was ascertained from routinely administered cognitive assessments, making use of natural language processing algorithms with established validity.
- However, dementia stage and hospitalisations were measured contemporaneously, therefore it is not appropriate to draw conclusions about causation.
- Reasons for hospitalisation were derived from the discharge diagnoses recorded for last episode of the hospital spell, and it was therefore not possible to distinguish complications that arose during the hospital stay from reasons for the initial admission.
- Data were obtained from single service provider in an urban catchment area, which may limit the generalisability of our findings to other settings.
- The analyses did not account for comorbidities, use of medications or institutional residence.

For peer review only

INTRODUCTION

Dementia represents a significant challenge for health services. For example, in the UK National Health Service (NHS) an estimated 25% of acute beds are occupied by people with dementia[1] and recent analyses have shown a 35% increase in the number of emergency hospital admissions for people with dementia in England over the past five years.[2] In 2016, the global economic costs caused by dementia approached $1 trillion, with the highest economic burden seen in Europe and North America.[3] Major contributing factors to costs include disease severity and higher rates of hospitalisation in people with dementia relative to older people without dementia.[4]

Whilst some hospitalisations are necessary and expected, others are potentially avoidable. Ambulatory care sensitive conditions (ACSCs) are conceptualised as chronic and acute health conditions such as heart failure, pneumonia, pyelonephritis and complications of diabetes, for which effective primary and community care may avoid the need for hospital admission by preventing the onset of these diseases, controlling the acute episodic illness, or managing the chronic condition more effectively.[5 6] ACSC-related admissions, for example, are used to assess how the NHS is performing in England.[7]

For peer review only - http://bmjopen.bmj.com/site/about/guidelines.xhtml

Preventing hospitalisation is of particular importance in people with dementia; hospitalisation is associated with poorer outcomes including prolonged hospital stays and increased risk of complications relative to people without dementia.[8-11] Further, hospitalisation itself can trigger distress, or confusion which can contribute to a decline in functioning creating new care challenges, or can intensify the existing burden of care.[12]

Even at early stages of cognitive impairment, a person's ability to make health care decisions can be altered, [13] including changes in health-seeking behaviours and management of existing conditions [14]. This could explain why the age-adjusted risk of potentially avoidable hospitalisations is greater in people with dementia than those without. [9 10] There is also some evidence suggesting that the risk of potentially avoidable hospitalisations is greater in people with later-stage dementia.[8 15] However, previous studies have been small, used non-representative convenience samples or relatively crude methods to evaluate cognitive deficits. Understanding which potentially avoidable causes of hospitalisation predominate at later stages of dementia can help to tailor healthcare needs in this population in order to prevent the need for hospitalisation.

For peer review only - http://bmjopen.bmj.com/site/about/guidelines.xhtml

In the present study, we used data drawn from a mental healthcare case register linked to national hospitalisation episodes, to describe ACSC-related hospitalisations, a proxy for potentially avoidable hospitalisations, amongst a large sample of patients with dementia. Specifically, we sought first to investigate whether ACSC-related hospital admission was associated with more advanced stages of dementia, and second to explore whether hospitalisations for specific ACSCs were associated with more advanced dementia stage.

For peer review only

For peer review only - http://bmjopen.bmj.com/site/about/guidelines.xhtml

METHODS

Study design

This study investigated acute general hospital admissions around the time of a given cognitive function score (index date) in a sample of patients with a clinical diagnosis of dementia using linked clinical datasets.

Data sources and sample

The Clinical Record Interactive Search (CRIS) data resource was used to identify dementia cases. CRIS provides research access to anonymised electronic health records (EHRs) from the South London and Maudsley NHS Foundation Trust (SLaM), a provider of mental healthcare, dementia assessment and management, for a south London catchment of >1.2 million residents; EHRs have been implemented across all SLaM services since 2006.[16] CRIS data are linked to Hospital Episode Statistics (HES), described below.

Routine diagnoses recorded in SLaM are structured according to the International Classification of Diseases 10th edition (ICD-10) coding system, and are supplemented in CRIS by a natural language processing (NLP) to abstract diagnoses and measures of cognitive impairment.[16 17] Hospital outcomes (hospital admissions and their causes) for patients with

For peer review only

dementia were obtained from the HES-CRIS linkage. HES contains details of all inpatient admissions at NHS hospitals in England.[18] Discharge diagnoses are also recorded as ICD-10 codes and are available for each hospitalisation episode. HES data up to 31st March 2013 were available at the time of analysis.

For peer review only

Study population

Patients with a diagnosis of dementia first recorded in SLaM between 1 January 2006 and 30th September 2012, and aged ≥65 years at diagnosis, were identified on CRIS. Dementia was defined on the presence of F00*, F01*, F02*or F03* ICD diagnosis codes recorded up to the sixth position in the structured data. Eligible patients were those who had at least one cognitive assessment (from structured data or free text using NLP) recorded: either a Mini Mental State Examination score (MMSE) or a cognitive impairment score from the Health of the Nation Outcome Scales (HoNOS). HoNOS is a clinician-rated instrument usually completed on a structured field within the EHR at first assessment, which contains subscales rated 0 (no problem) to 4 (severe/very severe problem); its cognitive scale correlates with MMSE measurement.[19] To exclude patients with potential mild cognitive impairment rather than

For peer review only - http://bmjopen.bmj.com/site/about/guidelines.xhtml

dementia, the sample was further restricted to those with an MMSE score of <28 or HoNOS cognitive impairment score >1. In addition, patients who were in active contact with acute hospital liaison services at the time of initial diagnosis were excluded, since these reflect cases where the recording of a dementia diagnosis might have been precipitated by a hospitalisation.

For each eligible patient, we randomly selected a single measure of cognition recorded in the patient's clinical record using a random number generator in Microsoft Excel; the date for the measure was assigned as the index date. This process was applied hierarchically: if several MMSE scores were recorded over time for a given patient, then one was randomly selected; for those individuals without an MMSE measurement, a random Health of the Nation Outcome Scales (HoNOS) cognitive impairment subscale was selected in the same manner. The randomly selected score for each patient was used to categorise the stage of dementia into mild, moderate or severe on the date of the cognitive assessment as described below.

Measurements

The following demographic information was obtained from CRIS: age at diagnosis of dementia, sex, ethnicity (White-European, other, not specified), dementia diagnostic group (Alzheimer's, vascular, mixed or other) and Index of Multiple Deprivation (IMD) score, a

For peer review only - http://bmjopen.bmj.com/site/about/guidelines.xhtml
For peer review only

widely used measure of relative neighbourhood deprivation.[20] IMD is derived from Census data and calculated for clusters of adjacent unit postcodes in England known as Lower layer Super Output Areas (LSOA), standard geographic units with around 1500 residents; a higher IMD score indicates a more deprived area.

To classify dementia stage at the index date, we used cut-offs similar to those used by others[21], defining MMSE scores of >20, 10-20, and <10, or a HoNOS cognitive impairment subscale score of 2, 3, and 4, as representing mild, moderate, or severe dementia, respectively.

Acute general hospital inpatient admissions were obtained for the 6-month period either side of the index date, i.e., over a 12-month period. Hospitalisations were defined from HES episodes, combining contiguous episodes (i.e., where start and end dates were on the same day); in the context of this analysis, an admission, or spell, is defined as a continuous period spent as a patient in hospital. Four-digit ICD-10 discharge diagnoses recorded during the last episode of each hospital spell were obtained (up to twenty diagnoses may be recorded as part of a hospital episode).

For peer review only - http://bmjopen.bmj.com/site/about/guidelines.xhtml
For peer review only

Ambulatory care sensitive conditions (ACSC) were identified based on the presence of specific ICD-10 codes and their position in the record, using a modification of the approach used by Bardsley *et al* and the UK Health and Social Care Information Centre; for example, angina is based on the presence of specific ICD-10 codes in the primary position, whereas pneumonia is defined based on the presence of a relevant code in any position.[5 6] Deviations from the published approach comprised the following: i) for the UTI category, we deemed cystitis to be relevant and therefore included relevant codes in this group; ii) we removed O15 (eclampsia) from the convulsions and epilepsy category as used by Bardsley. Each ACSC was specified individually as a binary variable to flag the presence of a hospitalisation with that specific condition. Given that diagnoses may not be recorded in the order of clinical importance in the real-world setting, as a sensitivity analysis, we relaxed the requirement for conditions which were based on a diagnosis in the primary position only, to include diagnoses in either of the first two positions. **Supplementary table 1** lists the ACSCs, the ICD codes and the position of the ICD code.

For peer review only

Additional covariates included binary variables to indicate an all-cause or any ACSC-related hospitalisation during the 12-month observation period. Time since first recorded dementia diagnosis (a proxy for disease duration) in SLaM at index date was also obtained.

Statistical analyses

Microsoft Excel was used for data management, and Stata 15·0 was used for statistical analyses. Patient characteristics were described by stage of dementia; normally distributed variables were presented as mean ± standard deviation (SD) and non-normally distributed variables as median and upper and lower quartiles. For the bivariate analysis, comparison of continuous variables between dementia stages (mild, moderate, severe) was evaluated using ANOVA, with the exception of number of inpatient admissions and time since presentation at SLaM which were evaluated by the Kruskal-Wallis test due to their non-normal distribution. For the relationship between each categorical variable and stage of dementia, a chi-squared test of association was performed, and percentage distributions presented.

To evaluate the association between any ACSC-related hospitalisation and contemporaneously measured stage of dementia, unadjusted, age-sex-adjusted and fully adjusted odds ratios (OR)

For peer review only - http://bmjopen.bmj.com/site/about/guidelines.xhtml

were obtained from generalised ordinal logistic regression models,[22] with dementia stage (mild, moderate, severe) as the dependant variable. Including dementia stage as the outcome variable was deemed to be the most efficient approach to evaluate ACSC-related hospitalisations and specific causes of ACSC-related admissions as it allowed us to use the efficient, flexible approach of stepwise model selection, to evaluate the importance of the relationship with other variables. Because our aim was to assess co-temporal associations between stage and hospitalisations rather than causality, we considered that whether dementia stage was modelled as a response, or as an explanatory variable, was not critical.

To investigate whether specific causes of ACSC-related admissions were associated with dementia stage, a backward stepwise model selection was used to build a multivariable generalised ordinal logistic regression model, with dementia stage as the dependant variable. We used the `gologit2` STATA command with the `autofit` option to selectively relax the model, removing the proportional odds assumption for any variable for which this assumption was violated.[22] Sex, time from first dementia record at index date (years) and age-group at index date were deemed clinically relevant and were therefore forced into the model (i.e., retained regardless of their p-value). Variables with p<0.1 in the bivariate analysis were

For peer review only - http://bmjopen.bmj.com/site/about/guidelines.xhtml

initially included in the stepwise model selection. During stepwise selection, variables with p <0.2 were retained in the model, and following exclusion of any variable, the variables already excluded were considered for re-inclusion. Collinearity was evaluated based on the Variance Inflation Factor (VIF) and tolerance for each variable; covariates with a VIF greater than 2.5 were removed one at a time, starting with variables with the largest VIF, and the impact on remaining covariates was assessed until no remaining variable had a VIF >2.5. Further, interactions between age-group and sex, and between age-group and dementia diagnostic group, were assessed for possible inclusion, based on the Akaike Information Criterion (AIC).

Patient and Public Involvement

Patients or the public were not involved in the design, or conduct, or reporting, or dissemination plans of our research.

For peer review only - http://bmjopen.bmj.com/site/about/guidelines.xhtml

RESULTS

In total, 5,294 patients with dementia met the study criteria and were included in the analysis sample. Almost two-thirds of the sample were female (N=3,393, 64.1%) and the mean age at the date of randomly chosen cognitive assessment was 82.5 years (SD 6.8). The proportions of patients with mild, moderate and severe dementia at these index dates were 37.3%, 46.4% and 16.1% respectively. Descriptive characteristics by stage of dementia are presented in **Table 1.**

Table 1: Descriptive analysis by dementia severity (N=5,294)

		Mild (N=1,979)	Moderate (N=2,461)	Severe (N=854)	All (N=5,294)	Test statistic [d.f] p value
**Age at index date N (%)**	65-74y	326 (16.5)	292 (11.87)	116 (13.58)	734 (13.86)	χ^2 [4] = 23.35 p <0.0001
	75-84y	892 (45.1)	1107 (44.98)	397 (46.49)	2396 (45.26)	
	85+	761 (38.5)	1062 (43.15)	341 (39.93)	2164 (40.88)	
**Mean age at index date (SD)**		81.89 (6.9)	82.9 (6.75)	82.44 (6.9)	82.45 (6.84)	F [2, 5291] = 11.96 p <0.0001
**Sex**	Female	1233 (62.3)	1615 (65.62)	545 (63.82)	3393 (64.09)	χ^2 [2]=5.29 p 0.071
	Male	746 (37.7)	846 (34.38)	309 (36.18)	1901 (35.91)	
**Ethnic group**	White-European	1614 (81.56)	1915 (77.81)	655 (76.7)	4184 (79.03)	χ^2 [4] = 9.54 p 0.008
	Other	337 (17.03)	502 (20.4)	172 (20.14)	1011 (19.1)	
	Not Specified	28	44	27	99	

For peer review only - http://bmjopen.bmj.com/site/about/guidelines.xhtml

		(1.41)	(1.79)	(3.16)	(1.87)	
**Deprivation score - mean (SD)**		26.02 (11.35)	27.46 (11.01)	28.1 (11.03)	27.02 (11.17)	F [2, 5260] = 13.75 p <0.0001
**Time (years) since first presentation at SLaM – median (IQR)**		0.31 (0.033 ,0.91)	0.31 (0.016 ,1.17)	0.31 (0.022 ,1.28)	0.31 (0.02 ,1.06)	H (2) = 1.04 p 0.596
**Dementia diagnostic group**	Alzheimer's	852 (43.05)	931 (37.83)	336 (39.34)	2119 (40.03)	χ^2 [6] = 25.81 p <0.0001
	Mixed	206 (10.41)	278 (11.3)	74 (8.67)	558 (10.54)	
	Vascular	385 (19.45)	602 (24.46)	197 (23.07)	1184 (22.36)	
	Other	536 (27.08)	650 (26.41)	247 (28.92)	1433 (27.07)	.

Test statistics: χ^2 Chi square test of association, F: One-way ANOVA, H: Kruskal-Wallis test.

d.f: degrees of freedom

All-cause and ACSC-related hospitalisations

Overall, 56.5% (N=2,993 of 5,294) of individuals analysed had at least one inpatient admission in the 6-month period either side of the index date, and this percentage was greater with more advanced dementia: 53.9% of patients with mild, 57.1% with moderate and 60.9% with severe dementia (χ^2 (d.f. 2) =12.47, $p = 0.002$) (**Table 2**).

Within the cohort, 22.5% had at least one ACSC-related hospitalisation (N=1,192/5,294) which accounted for almost 40% of patients with any hospital admission (N=1,192/2,993). ACSC-related hospitalisations were observed in 36.2%, 42.0% and 41.5% of hospitalised patients with mild, moderate and severe dementia respectively (χ^2 (d.f. 2) =9.25 $p = 0.01$). The most common ACSC was UTI/pyelonephritis (9.8% of hospitalised patients) followed by

For peer review only

pneumonia (7.1%), both of which tended to be more frequent with more severe dementia (p <0.0001 for both conditions). The mean number of ACSC admissions amongst hospitalised patients was highest in those with moderate (0.67, SD 1.04) compared to mild or severe dementia (0.57, SD 1.01 and 0.58, SD 0.85, F [2, 2990] = 3.33, p = 0.036).

For peer review only

For peer review only - http://bmjopen.bmj.com/site/about/guidelines.xhtml

Table 2: Hospital admissions by severity of dementia (N=5,294)

	Mild (N=1979)	Moderate (N=2461)	Severe (N=854)	All (N=5294)	Test statistic [d.f] p value
Inpatient admission +/- 6 months of index date, N (%)	1067 (53.92)	1406 (57.13)	520 (60.89)	2993 (56.54)	χ^2 [2] = 12.47 p 0.002
Number of all cause admissions per patient – Median (lower and upper quartile)*	2 (1,3)	2 (1,3)	2 (1,3)	2 (1,3)	H [2] = 3.42 p 0.181
Ambulatory care sensitive conditions N (% of all patients)					
Angina	27 (1.36)	28 (1.14)	5 (0.59)	60 (1.13)	χ^2 [2] = 3.2 p 0.199
Asthma	2 (0.1)	2 (0.08)	1 (0.12)	5 (0.09)	χ^2[2] = 0.10 p 0.951
Cellulitis	19 (0.96)	27 (1.1)	9 (1.05)	55 (1.04)	χ^2 [2] = 0.20 p 0.904
Congestive heart failure	28 (1.41)	38 (1.54)	10 (1.17)	76 (1.44)	χ^2 [2] = 0.63 p 0.729
Convulsion/epilepsy	16 (0.81)	23 (0.93)	12 (1.41)	51 (0.96)	χ^2 [2] = 2.26 p 0.322
COPD	28 (1.41)	40 (1.63)	8 (0.94)	76 (1.44)	χ^2 [2] = 2.13 p 0.344
Dehydration	8 (0.4)	20 (0.81)	7 (0.82)	35 (0.66)	χ^2[2] = 3.18 p 0.204
Dental	9 (0.45)	10 (0.41)	1 (0.12)	20 (0.38)	χ^2 [2] = 1.91 p 0.385
Diabetes complications	30 (1.52)	48 (1.95)	11 (1.29)	89 (1.68)	χ^2 [2] = 2.205 p 0.332
ENT infections	5 (0.25)	2 (0.08)	1 (0.12)	8 (0.15)	χ^2 [2] = 2.21 p 0.331
Flu	-	-	-	-	-
Gangrene	-	-	-	-	-
Gastroenteritis	21 (1.06)	20 (0.81)	3 (0.35)	44 (0.83)	χ^2 [2] = 3.67 p 0.16
Hypertension	3 (0.15)	3 (0.12)	-	6 (0.11)	χ^2 [2] = 1.24 p 0.538
Iron deficiency anaemia	6 (0.3)	15 (0.61)	2 (0.23)	23 (0.43)	χ^2 [2] = 3.32 p 0.19

For peer review only - http://bmjopen.bmj.com/site/about/guidelines.xhtml

**Other vaccine preventable**	2 (0.1)	-	-	2 (0.04)	χ^2 [2] = 3.35 p 0.187
**Perforated/bleeding ulcer**	-	3 (0.12)	3 (0.35)	6 (0.11)	χ^2 [2] = 6.53 p 0.038
**Pneumonia**	103 (5.2)	201 (8.17)	70 (8.2)	374 (7.06)	χ^2 [2] = 16.653 p <0.0001
**UTI pyelonephritis**	155 (7.83)	255 (10.36)	111 (13)	521 (9.84)	χ^2 [2] = 19.34 p <0.0001
**Any ACSC admission N (%)**					
**overall**	386 (19.5)	590 (23.97)	216 (25.29)	1192 (22.52)	χ^2 [2] = 17.06 p <0.0001
**amongst those with an inpatient admission**	386 (36.18)	590 (41.96)	216 (41.54)	1192 (39.83)	χ^2 [2] = 9.25 p 0.010
**Mean number of ACSC admissions per patient (SD)**					
**amongst those with an inpatient admission**	0.57 (1.01)	0.67 (1.04)	0.58 (0.85)	0.62 (1)	F [2 2990] = 3.33 p 0.036

* amongst hospitalised

Test statistics: χ^2 Chi square test, F: One-way ANOVA, H: Kruskal-Wallis test

d.f: degrees of freedom

For peer review only

For peer review only - http://bmjopen.bmj.com/site/about/guidelines.xhtml

Associations between any ACSC-related hospitalisation and dementia stage

Any ACSC-related hospitalisation was significantly associated with more advanced dementia, i.e., an OR significantly greater than 1 for moderate/severe vs. mild, or severe vs. mild/moderate, in the unadjusted model (unadjusted OR 1.28, 95% CI 1.14, 1.45). The association remained significant in the fully adjusted model which accounted for age, sex, time since first presentation on SLaM, ethnic-group and neighbourhood deprivation (OR 1.27, 95% CI 1.12, 1.44 for both moderate/severe vs. mild and severe vs. mild/moderate) (**Table 3**).

Table 3: Unadjusted and adjusted ORs and 95% CIs for any ACSC-related admission by stage of dementia

	**Severe/moderate vs. mild or Severe vs. mild/moderate□ OR (95% CI)**
**Unadjusted**	1.28 (1.14, 1.45)
**Age, sex adjusted**	1.27 (1.13, 1.44)
**Fully adjusted***	1.27 (1.12, 1.44)

*Age-group, sex, time since first presentation on SLaM, ethnicity and IMD

□From a generalised ordinal logistic regression model: the same odds ratio applies to both comparisons as the proportional odds assumption is not violated.

Exploratory analysis: associations between specific ACSC-related hospitalisations and dementia stage

Variables included in the multivariable model after stepwise selection are summarised in **Table 4**. The goodness of fit of the final model, as measured by the likelihood ratio chi-square test,

For peer review only - http://bmjopen.bmj.com/site/about/guidelines.xhtml

demonstrated that the model as a whole fitted significantly better than the null model (i.e. a model with no predictors; LR Chi square 144.6, p <0.0001). The output from the stepwise-selected regression model is presented in **Table 4**. The proportional odds assumption was violated for age-group, and dementia diagnostic group, meaning that the effect of these explanatory variables was not the same at the two dementia-severity boundaries.

The model indicated that, conditional on other explanatory variables, a greater time since first presentation at SLaM, higher IMD score (i.e., residence in a more deprived area) and “other” ethnicity were associated with greater odds of more severe dementia. Greater age was associated with greater odds of moderate/severe dementia relative to mild, but not of severe dementia relative to mild/moderate.

Considering specific conditions, perforated/bleeding ulcer, UTI/pyelonephritis and pneumonia hospitalisations were all significantly associated with greater odds of more advanced dementia, i.e., moderate/severe relative to mild or severe relative to mild/moderate. Although a strong association between perforated/bleeding ulcer and dementia stage (OR 7.01 p 0.01) was observed, it is important to note that in terms of absolute frequency this condition was

For peer review only

uncommon, being recorded in only 6 individuals. UTI/pyelonephritis and pneumonia were also associated with higher odds of more severe dementia (OR 1.38 p <0.0001, OR 1.38 p 0.002 respectively); this was consistent across dementia severity categories, i.e., the ORs and p-values apply to both cut-points. Gastroenteritis-related admissions had an inverse relationship with dementia-stage, namely that admission was associated with a lower odds of advanced dementia stages: however, this was of borderline significance (OR 0.57 p 0.06).

Table 4: Results from stepwise-selected partial generalised ordinal logistic regression model for severity of dementia (N=5,294)*

Explanatory variable	Moderate/severe vs. mild dementia		Severe vs. mild/moderate Dementia	
	OR (95% CI)	p	OR (95% CI)	p
Sex (male reference)	1.07 (0.96, 1.2)	0.203	1.07 (0.96, 1.2)	0.203
Time since first presentation at SLaM	1.13 (1.08, 1.19)	<0.0001	1.13 (1.08, 1.19)	<0.0001
Age group at index date (65-74 years reference)				
	1.36 (1.15, 1.62)	<0.0001	1.04 (0.82, 1.31)	0.763
	1.53 (1.28, 1.83)	<0.0001	1.01 (0.79, 1.28)	0.949
Deprivation score	1.01 (1.01, 1.02)	<0.0001	1.01 (1.01, 1.02)	<0.0001
Dementia diagnostic group (AD reference)				
Vascular	1.34 (1.15, 1.56)	<0.0001	1.02 (0.84, 1.24)	0.825
Mixed	1.16 (0.95, 1.4)	0.145	0.8 (0.61, 1.05)	0.114
Other	1.12 (0.98, 1.27)	0.099	1.12 (0.98, 1.27)	0.099

For peer review only

For peer review only - http://bmjopen.bmj.com/site/about/guidelines.xhtml

Ethnic group (European reference)				
Other	1.2 (1.05, 1.37)	0.009	1.2 (1.05, 1.37)	0.009
**Pneumonia**	1.38 (1.13, 1.7)	0.002	1.38 (1.13, 1.7)	0.002
**Perforated/bleeding ulcer**	7.01 (1.56, 31.57)	0.011	7.01 (1.56, 31.57)	0.011
**Dehydration**	1.71 (0.92, 3.17)	0.09	1.71 (0.92, 3.17)	0.09
**Gastroenteritis**	0.57 (0.32, 1.02)	0.059	0.57 (0.32, 1.02)	0.059
**UTI pyelonephritis**	1.38 (1.15, 1.65)	<0.0001	1.38 (1.15, 1.65)	<0.0001

* Proportional odds assumption violated for age and dementia diagnostic group. Note that where it is not violated, the same odds ratio applies to both comparisons.

For a continuous or an ordinal variable, the odds ratio is calculated relative to a one-unit change in the variable. For ACSCs, the reference category is absence of the condition recorded.

ACSC: ambulatory care sensitive condition

Goodness of fit: Likelihood ratio test - LR Chi square 144.6, p <0.0001

Sensitivity analysis (expanded ACSC definition)

In addition to perforated/bleeding ulcer (OR 6.92), pneumonia (OR 1.38) and UTI pyelonephritis (OR 1.49), in the sensitivity analysis where the ACSC definition was relaxed, convulsion/epilepsy (OR 1.58) was also found to be significantly associated with more severe dementia (**Supplementary table 2**).

For peer review only - http://bmjopen.bmj.com/site/about/guidelines.xhtml

DISCUSSION

We evaluated all-cause and potentially avoidable-causes of hospitalisation around the times of randomly selected cognitive function assessments in people with clinically diagnosed dementia, modelling mild vs. moderate vs. severe dementia at the index dates in question. Over half of our sample had a hospitalisation of any kind during a 12-month period of evaluation, and almost a quarter had an ACSC-related hospital admission, which accounted for nearly 40% of hospitalised patients. The proportions of patients with at least one hospitalisation overall, or with one ACSC-related hospitalisation, were greater in the groups with more advanced dementia. The odds of being in a more advanced stage was almost 1.3-fold higher for those with an ACSC-related admission after adjusting for demographics, time from first dementia record at index date (a proxy for disease duration) and neighbourhood deprivation.

Around 6% of community-dwelling individuals with dementia in a US Veterans Health Administration database had at least one ACSC admission during a 12-month period; however, Medicare facility-use was not included, and hospitalisations may thus have been underestimated.[23] In another study, an average of 28% of Medicare beneficiaries per year aged ≥65 years with dementia participating in the Washington Heights-Inwood Columbia Aging

For peer review only

For peer review only - http://bmjopen.bmj.com/site/about/guidelines.xhtml

Project (WHICAP), had an ACSC-related admission,[8] which was more consistent with our own findings.

After adjusting for a range of clinical and demographic variables, we found that an ACSC-related admission remained associated with more advanced dementia. Hospitalisations with UTI/pyelonephritis, pneumonia and perforated/bleeding ulcer recorded as part of an admission were associated with more advanced dementia in our study. Although these have been reported as common causes of admission in people with dementia relative to those without dementia,[24-26] there is sparse literature on relationships with dementia severity. A study of Medicare beneficiaries with dementia found that that worse cognitive status was associated with higher risks of hospitalisations for diabetes, pneumonia, hypertension, and dehydration, and that worse functional status was associated with higher risks of hospitalisation for diabetes and UTI, marginally higher for dehydration, but lower for hypertension.[8]

UTI/pyelonephritis was recorded in 17.4% of hospitalised patients with dementia in our sample, and its presence was associated with more severe dementia. UTIs in more severe dementia may reflect worse immune function,[27] problems with mobility associated with urinary

For peer review only - http://bmjopen.bmj.com/site/about/guidelines.xhtml

incontinence and subsequent infection,[28] and inactivity or dehydration.[29] Consistently, we also observed a suggestion of association with dehydration in more severe dementia, although this was of not significant (p = 0.09).

Hospitalised pneumonia was common in our sample (12.5% of hospitalised patients with dementia) and was associated with more advanced dementia, as has been described by others.[30 31] Uptake of the pneumococcal vaccine in the over-65 age-group is relatively high in the UK (69.5% of the primary care population)[32]: this suggests that pneumonia in our sample may be due to other bacterial or viral causes, or may be occurring as a result of an impaired immune system, dysphagia or urinary incontinence, which are common in, and associated with a higher risk of pneumonia in, the elderly.[33 34] Furthermore, it was not possible to determine whether instances of pneumonia identified in our sample were acquired within hospital as part of the inpatient episode, or in the community prior to hospitalisation.

Although only 0.2% of patients in our sample had a hospitalisation with perforated/bleeding ulcer recorded, this condition was strongly associated with more severe dementia at the index date. Gastrointestinal bleeding is a frequent indication for hospitalisation in the elderly [35], and

For peer review only - http://bmjopen.bmj.com/site/about/guidelines.xhtml

there is evidence that the incidence of peptic ulcer disease is higher in people with dementia compared to those without, after accounting for age, sex, and comorbidity.[10 26] We are not aware of any studies investigating differences by dementia stage on risk of gastrointestinal bleeding; understanding differences in the use of and adherence to medications such proton pump inhibitors which are indicated for the management and/or prevention of GI bleeding, by dementia severity, could be useful to understand whether such risk differences may explain our observations.

Strengths and limitations

Our study evaluated hospitalisations in a large sample of clinically diagnosed cases of dementia where cognitive assessment is performed as part of routine clinical care. Linkage of our clinical dataset to a national hospitalisation database benefited from near-complete outcome ascertainment, since hospitalisation is free at the point of delivery in the UK context. We also benefited from information allowing differentiation between mild, moderate and severe dementia. Considering limitations, the analysed sample was from a single urban and suburban catchment and included only cases with dementia diagnosed in specialist services; however, the estimated proportion of people with dementia in the SLaM catchment who receive a

For peer review only

specialist diagnosis is relatively high at 75.2%.[36] Considering reasons for hospitalisation, these were derived from the discharge diagnoses recorded for last episode of the hospital spell, and it was not possible to distinguish complications that arose during the hospital stay from reasons for the initial admission. Considering residual confounding, this analysis did not account for comorbidity burden, medication use or residence type, all of which are potentially associated with hospitalisations and dementia severity,[9 11 23 37] nor did it account for prior healthcare utilisation, functional ability,[37] and psychiatric symptoms. Patients with severe dementia were under-represented in our sample and the time since first diagnosis in SLaM in these patients was relatively short despite the extent of cognitive impairment; it is therefore possible that our patients with severe dementia are generally those presenting late in their disease course, and not representative of the general advanced-dementia population. Our analysis evaluated associations between hospitalisation patterns and contemporaneously measured severity of dementia, so it is not appropriate to draw conclusions about causation. In addition, we evaluated hospitalisation outcomes up to 6-months after a cognitive measure and did not attempt to account for survival effects. Mortality increases with severity of dementia[31], and is a competing risk for hospitalisation, although the relatively short timeframe evaluated should mitigate the effects of this.

For peer review only

Conclusions

Our study adds to a small but increasing body of evidence on the relationship between dementia and potentially avoidable hospitalisations. Despite the clinical and economic burden, we are not aware of any studies in the UK setting that describe potentially avoidable hospitalisations in patients with dementia. We show that potentially avoidable hospitalisations are common in people with dementia, and in particular in those with more advanced dementia; these findings are of importance given that hospitalisation is a significant element in the cost of dementia care and that the number of people with dementia is increasing. Greater attention to the extent of cognitive impairment, and not only the presence of a dementia diagnosis, is needed in order to prevent hospitalisations. However, more work is clearly needed to better understand factors contributing to the high rates of ACSC-related hospitalisation, including the management of ACSCs in the primary care setting. Reducing admissions for ACSCs represents an opportunity to improve both the quality and efficiency of care in healthcare systems.

Ethics statements

Patient consent for publication

Not required.

For peer review only - http://bmjopen.bmj.com/site/about/guidelines.xhtml

Ethics approval

The Oxford Research Ethics Committee C (reference 18/SC/0372) approved CRIS at the Maudsley as a data resource for secondary analysis.

Author contributions

UG was responsible for the design, analysis, interpretation and drafting of the manuscript. GP contributed to the drafting of the manuscript. NG provided statistical input and contributed to the drafting of the manuscript. RS contributed to the design, interpretation and drafting of the manuscript

Funding

This work was supported by NIHR Biomedical Research Centre at the South London and Maudsley NHS Foundation Trust and King's College London. RS is additionally part-funded by a Medical Research Council Mental Health Data Pathfinder Award to King's College London, and an NIHR Senior Investigator Award

Role of the funding source

For peer review only

The funder of the study had no role in the study design, data collection, data analysis, data interpretation, or writing of the report. UG, GP and RS had access to the anonymised data. The corresponding author had the final responsibility for the decision to submit for publication.

Declaration of Competing Interest

UG and NG are employees of GSK, hold stock and receive a salary from GSK. RS has received research funding in the last 5 years from Roche, Janssen, Takeda and GSK.

Acknowledgments

The authors would like to acknowledge Hitesh Shetty for his contributions to the study.

Data sharing

Because of their nature and to comply with their ethical approval, CRIS data are required to remain within the firewall of the South London and Maudsley NHS Foundation Trust (SLaM). Access to the data used for this study can be facilitated by the CRIS Oversight Committee on application and with appropriate SLaM affiliation, details of which can be obtained from cris.administrator@slam.nhs.uk.

References

1. Dementia Statistics Hub. Dementia Statistics. Hospitals online [Available from: https://www.dementiastatistics.org/statistics/hospitals/ accessed 19/03/2021 2021.
2. Alzheimer's Society. Analysis of NHS England's Hospital Episode Statistics dataset 2012/13 to 2017/18: Alzheimer's Society, 2020.
3. Xu JZ, Y.; Qiu, C.; Cheng, F. Global and regional economic costs of dementia: a systematic review. *The Lancet* 2017;390:S47. doi: 10.1016/S0140-6736(17)33185-9
4. Prince MK, M.; Guerchet, M.; McCrone, P.; Prina, M.; Comas-Herrera, A.; Wittenberg, R.; Adelaja, B.; Hu, B.; King, D.; Rehill, A.; Salimkumar, D.;. Dementia UK Update, 2014.

For peer review only - http://bmjopen.bmj.com/site/about/guidelines.xhtml

5. NHS Digital. Enhancing quality of life for people with long-term conditions. In: Team CI, ed. UK: Health and Social Care Information Centre (HSIC), 2017.
6. Bardsley M, Blunt I, Davies S, et al. Is secondary preventive care improving? Observational study of 10-year trends in emergency admissions for conditions amenable to ambulatory care. *BMJ Open* 2013;3(1) doi: 10.1136/bmjopen-2012-002007 [published Online First: 2013/01/05]
7. Blunt I. Focus on preventable admissions. Trends in emergency admissions for ambulatory care sensitive conditions, 2001 to 2013. QualityWatch Focus, 2013.
8. Zhu CW, Cosentino S, Ornstein K, et al. Use and cost of hospitalization in dementia: Longitudinal results from a community-based study. *International Journal of Geriatric Psychiatry* 2015;30(8):833-41. doi: 10.1002/gps.4222
9. Bynum JPW, Rabins PV, Weller W, et al. The Relationship between a Dementia Diagnosis, Chronic Illness, Medicare Expenditures, and Hospital Use. *Journal of the American Geriatrics Society* 2004;52(2):187-94. doi: 10.1111/j.1532-5415.2004.52054.x
10. Phelan EA, Borson S, Grothaus L, et al. Association of incident dementia with hospitalizations. *JAMA - Journal of the American Medical Association* 2012;307(2):165-72. doi: 10.1001/jama.2011.1964
11. Lin PJ, Fillit HM, Cohen JT, et al. Potentially avoidable hospitalizations among Medicare beneficiaries with Alzheimer's disease and related disorders. *Alzheimer's and Dementia* 2013;9(1):30-38. doi: 10.1016/j.jalz.2012.11.002
12. Alzheimer's Association. Reducing potentially preventable hospitalisations for people living with Alzheimer's and other dementias 2017 [Available from: https://pdfs.semanticscholar.org/9448/37faa2cb818b66264a51d4a346aa8c1a0f93.pdf.
13. Han SD, Boyle PA, James BD, et al. Mild cognitive impairment is associated with poorer decision-making in community-based older persons. *J Am Geriatr Soc* 2015;63(4):676-83. doi: 10.1111/jgs.13346 [published Online First: 2015/04/09]
14. Ibrahim JE, Anderson LJ, MacPhail A, et al. Chronic disease self-management support for persons with dementia, in a clinical setting. *J Multidiscip Healthc* 2017;10:49-58. doi: 10.2147/JMDH.S121626 [published Online First: 2017/02/10]
15. Lin PJ, Rane PB, Fillit HM, et al. National estimates of potentially avoidable hospitalizations among medicare beneficiaries with Alzheimer's disease and related dementias. *Alzheimer's and Dementia* 2016;12(7):P253-P54.
16. Perera G, Broadbent M, Callard F, et al. Cohort profile of the South London and Maudsley NHS Foundation Trust Biomedical Research Centre (SLaM BRC) Case Register: current status and recent enhancement of an Electronic Mental Health Record-derived data resource. *BMJ open* 2016;6(3):e008721.
17. Jackson RG, Patel R, Jayatilleke N, et al. Natural language processing to extract symptoms of severe mental illness from clinical text: the Clinical Record Interactive Search Comprehensive Data Extraction (CRIS-CODE) project. *BMJ open* 2017;7(1):e012012.
18. NHS. Hospital Episode Statistics (HES) 2019 [Available from: https://digital.nhs.uk/data-and-information/data-tools-and-services/data-services/hospital-episode-statistics accessed 13/01/2019 2019.
19. Canuto A, Weber K, Gold G, et al. Structured assessment of mental health status in psychogeriatrics: validity of the French HoNOS65+. *Can J Psychiatry* 2007;52(1):37-45. doi: 10.1177/070674370705200107 [published Online First: 2007/04/21]

For peer review only

20. UK Government. The English Indices of Deprivation 2019. In: Ministry of Housing CaLG, ed., 2019.
21. NICE. Donepezil, galantamine, rivastigmine and memantine for the treatment of dementia. Health technology appraisal 2018 update. In: NICE, ed. 2018 ed, 2018.
22. Williams R. Understanding and interpreting generalized ordered logit models. *The Journal of Mathematical Sociology* 2016;40:7-20.
23. Maust DT, Kim HM, Chiang C, et al. Predicting Risk of Potentially Preventable Hospitalization in Older Adults with Dementia. *Journal of the American Geriatrics Society* 2019;67(10):2077-84. doi: 10.1111/jgs.16030
24. Natalwala A, Potluri R, Uppal H, et al. Reasons for hospital admissions in dementia patients in Birmingham, UK, during 2002-2007. *Dementia and Geriatric Cognitive Disorders* 2008;26(6):499-505. doi: 10.1159/000171044
25. Rao A, Suliman A, Vuik S, et al. Outcomes of dementia: Systematic review and meta-analysis of hospital administrative database studies. *Arch Gerontol Geriatr* 2016;66:198-204. doi: 10.1016/j.archger.2016.06.008
26. Hsu CC, Hsu YC, Chang KH, et al. Association of Dementia and Peptic Ulcer Disease: A Nationwide Population-Based Study. *Am J Alzheimers Dis Other Demen* 2016;31(5):389-94. doi: 10.1177/1533317515617546 [published Online First: 2016/01/24]
27. Janssens JP. Pneumonia in the elderly (geriatric) population. *Curr Opin Pulm Med* 2005;11(3):226-30. [published Online First: 2005/04/09]
28. Schnelle JF, Leung FW. Urinary and fecal incontinence in nursing homes. *Gastroenterology* 2004;126(1 Suppl 1):S41-7. [published Online First: 2004/02/24]
29. Palmer JL, Metheny NA. Preventing aspiration in older adults with dysphagia. *Am J Nurs* 2008;108(2):40-8; quiz 49. doi: 10.1097/01.NAJ.0000308961.99857.33 [published Online First: 2008/01/30]
30. Knox S, Downer B, Haas A, et al. Dementia Severity Associated With Increased Risk of Potentially Preventable Readmissions During Home Health Care. *Journal of the American Medical Directors Association* 2019 doi: 10.1016/j.jamda.2019.09.012
31. Sachs GA, Carter R, Holtz LR, et al. Cognitive impairment: an independent predictor of excess mortality: a cohort study. *Ann Intern Med* 2011;155(5):300-8. doi: 10.7326/0003-4819-155-5-201109060-00007 [published Online First: 2011/09/07]
32. NHS. Pneumococcal Polysaccharide Vaccine (PPV) coverage report, England, April 2018 to March 2019. In: England PH, ed., 2019.
33. Marrie TJ. Community-acquired pneumonia in the elderly. *Clinical Infectious Diseases* 2000;31(4):1066-78. doi: 10.1086/318124
34. Chong CP, Street PR. Pneumonia in the elderly: a review of the epidemiology, pathogenesis, microbiology, and clinical features. *South Med J* 2008;101(11):1141-5; quiz 32, 79. doi: 10.1097/SMJ.0b013e318181d5b5 [published Online First: 2008/12/18]
35. Yachimski PS, Friedman LS. Gastrointestinal bleeding in the elderly. *Nat Clin Pract Gastroenterol Hepatol* 2008;5(2):80-93. doi: 10.1038/ncpgasthep1034 [published Online First: 2008/02/07]
36. NHS England. Dementia diagnosis monthly workbook: Secondary dementia diagnosis monthly workbook 2017, 2017.

For peer review only - http://bmjopen.bmj.com/site/about/guidelines.xhtml

37. Shepherd H, Livingston G, Chan J, et al. Hospitalisation rates and predictors in people with dementia: A systematic review and meta-analysis. *BMC Medicine* 2019;17(1) doi: 10.1186/s12916-019-1369-7

For peer review only

For peer review only - http://bmjopen.bmj.com/site/about/guidelines.xhtml

For peer review only

For peer review only - http://bmjopen.bmj.com/site/about/guidelines.xhtml

Supplementary Table 1: Ambulatory care sensitive conditions*

ACSC	ICD code position		ICD-10 codes
	Main analysis	Sensitivity analysis	
**Angina**	Principal	1º or 2º	I20, I200, I201, I208, I209, I240, I248, I249, I25, I250, I251, I252, I253, I254, I255, I256, I258, I259
**Asthma**	Principal	1º or 2º	J45, J450, J451, J458, J459, J46, J46X
**Cellulitis**	Principal	1º or 2º	L03, L030, L031, L032, L033, L038, L039, L04, L040, L041, L042, L043, L048, L049, L08, L080, L081, L088, L089, L88, L88X, L980, L983
**Congestive heart failure**	Principal	1º or 2º	I110, I13, I130, I131, I132, I139, I50, I500, I501, I509, J81, J81X
**Convulsions and epilepsy**	Principal	1º or 2º	G40, G400, G401, G402, G403, G404, G405, G406, G407, G408, G409, G41, G410, G411, G412, G418, G419, O15, R56, R560, R568
**COPD**	Principal	1º or 2º	J20, J41, J410, J411, J418, J42, J42X, J43, J430, J431, J432, J438, J439, J44, J440, J441, J448, J449, J47, J47X
**Dehydration**	Principal	1º or 2º	E86, E86X
**Dental conditions**	Principal	1º or 2º	A690, K02, K020, K021, K022, K023, K024, K028, K029, K03, K030, K031, K032, K033, K034, K035, K036, K037, K038, K039, K04, K040, K041, K042, K043, K044, K045, K046, K047, K048, K049, K05, K050, K051, K052, K053, K054, K055, K056, K06, K060, K061, K062, K068, K069, K08, K080, K081, K082, K083, K088, K089, K09, K090, K091, K092, K098, K099, K12, K120, K121, K122, K13, K130, K131, K132, K133, K134, K135, K136, K137
**Diabetes complications**	Any	Any	E10, E100, E101, E102, E103, E104, E105, E106, E107, E108, E11, E110, E111, E112, E113, E114, E115, E116, E117, E118, E12, E120, E121, E122, E123, E124, E125, E126, E127, E128, E13, E130, E131, E132, E133, E134, E135, E136, E137, E138, E14, E140, E141, E142, E143, E144, E145, E146, E147, E148
**Ear, nose and throat infections**	Principal	1º or 2º	H66, H660, H661, H662, H663, H664, H669, H67, H670, H671, H678, J02, J020, J028, J029, J03, J030, J038, J039, J06, J060, J068, J069, J312
**Gangrene**	Any	Any	R02
**Gastroenteritis**	Principal	1º or 2º	K522, K528, K529
**Hypertension**	Principal	1º or 2º	I10, I10X, I119, I129, I139
**Influenza**	Any	Any	J10, J100, J101, J108, J11, J110, J111, J118
**Iron deficiency anaemia**	Principal	1º or 2º	D50, D501, D508, D509
**Nutritional deficiencies**	Principal	1º or 2º	E40, E40X, E41, E41X, E42, E42X, E43, E43X, E55, E550, E559, E64, E640, E641, E642, E643, E648, E649
**Nutritional deficiencies OR other nutritional deficiency anaemia**	Principal	1º or 2º	D51, D510, D511, D512, D513, D518, D519
**Other vaccine preventable**	Any	Any	A35, A35X, A36, A360, A361, A362, A363, A368, A369, A37, A370, A390, A80, A801, A802, A803, A804, A809, B05, B050, B051, B052, B053, B054, B058, B059, B06, B060, B068, B069, B16, B160, B161, B162, B169, B180, B181, B26, B260, B261, B262, B263, B268, B269, G000, G001, M010, M014
**Pelvic inflammatory disease**	Principal	1º or 2º	N70, N700, N701, N709, N73, N730, N731, N732, N733, N734, N735, N736, N738, N739, N74, N740, N741, N742, N743, N744, N748
**Perforated/bleeding ulcer**	Principal	1º or 2º	K25, K250, K251, K252, K254, K255, K256, K26, K260, K261, K262, K264, K265, K266, K27, K270, K271, K272, K274, K275, K276, K28, K280, K281, K282, K284, K286
**Pneumonia**	Any	Any	J13, J14, J153, J154, J157, J159, J168, J18, J180, J181, J182, J188, J189
**Tuberculosis**	Principal	1º or 2º	A15, A150, A151, A152, A153, A154, A155, A156, A157, A158, A159, A16, A160, A161, A162, A163, A164, A165, A167, A168, A169, A19, A190, A191, A192, A198, A199
**Urinary tract infection /Pyelonephritis**	Principal	1º or 2º	N10, N10X, N11, N110, N111, N118, N119, N12, N12X, N136, N30, N300, N301, N302, N303, N304, N308, N309, N390

ACSC: ambulatory care sensitive condition

*This table was created by the authors, however, the ACSCs, the respective ICD-10 codes and their position were initially based on those published by Bardsley *et al*, 2013. Codes were augmented with those published by the UK Health and Social Care Information Centre and by the authors. Deviations from the approach of Bardsley *et al* comprised of: i) cystitis codes were added to the UTI category; ii) O15 (eclampsia) was removed from the convulsions and epilepsy category. The approach used in the sensitivity was determined by the authors.

For peer review only

Supplementary Table 2: Results from stepwise-selected generalised ordinal logistic regression model for severity of dementia (sensitivity analysis *)

	Moderate/severe vs. mild dementia		Severe vs. mild/moderate dementia	
	**OR (95% CI)**	**p**	**OR (95% CI)**	**p**
**Sex (male reference)**	1.09 (0.97, 1.21)	0.141	1.09 (0.97, 1.21)	0.141
**Time since first presentation at SLaM**	1.13 (1.08, 1.18)	<0.001	1.13 (1.08, 1.18)	<0.001
**Age group at index date**	1.21 (1.11, 1.31)	<0.001	0.99 (0.89, 1.11)	0.856
**Ethnic group (European reference)**				
	1.21 (1.06, 1.38)	0.005	1.21 (1.06, 1.38)	0.005
**Dementia diagnostic group (AD reference)**				
Vascular	1.33 (1.15, 1.56)	<0.001	1.02 (0.85, 1.24)	0.816
Mixed	1.15 (0.95, 1.4)	0.163	0.8 (0.61, 1.05)	0.107
Other	1.11 (0.98, 1.27)	0.112	1.11 (0.98, 1.27)	0.112
**Deprivation score**	1.12 (1.06, 1.17)	<0.001	1.12 (1.06, 1.17)	<0.001
**Convulsions**	1.58 (0.96, 2.58)	0.07	1.58 (0.96, 2.58)	0.07
**Iron deficiency anaemia**	1.23 (0.63, 2.4)	0.542	0.25 (0.06, 1.05)	0.058
**Perforated/bleeding ulcer**	6.92 (1.56, 30.77)	0.011	6.92 (1.56, 30.77)	0.011
**Pneumonia**	1.38 (1.12, 1.69)	0.002	1.38 (1.12, 1.69)	0.002
**UTI pyelonephritis**	1.29 (1.1, 1.51)	0.002	1.29 (1.1, 1.51)	0.002

Goodness of fit: LR Chi square 143.3, *p*<0.0001
ACSC: ambulatory care sensitive condition
*Criteria were relaxed to include diagnoses in either of the first two positions for ACSCs that were initially defined based on a diagnosis in the primary position only.

For peer review only

STROBE 2007 (v4) checklist of items to be included in reports of observational studies in epidemiology*
Checklist for cohort, case-control, and cross-sectional studies (combined)

Section/Topic	Item #	Recommendation	Reported on page #
**Title and abstract**	1	(*a*) Indicate the study's design with a commonly used term in the title or the abstract	1
		(*b*) Provide in the abstract an informative and balanced summary of what was done and what was found	2
**Introduction**			
Background/rationale	2	Explain the scientific background and rationale for the investigation being reported	5
Objectives	3	State specific objectives, including any pre-specified hypotheses	5
**Methods**			
Study design	4	Present key elements of study design early in the paper	6
Setting	5	Describe the setting, locations, and relevant dates, including periods of recruitment, exposure, follow-up, and data collection	6
Participants	6	(*a*) *Cohort study*—Give the eligibility criteria, and the sources and methods of selection of participants. Describe methods of follow-up<br>*Case-control study*—Give the eligibility criteria, and the sources and methods of case ascertainment and control selection. Give the rationale for the choice of cases and controls<br>*Cross-sectional study*—Give the eligibility criteria, and the sources and methods of selection of participants	6
		(*b*) *Cohort study*—For matched studies, give matching criteria and number of exposed and unexposed<br>*Case-control study*—For matched studies, give matching criteria and the number of controls per case	
Variables	7	Clearly define all outcomes, exposures, predictors, potential confounders, and effect modifiers. Give diagnostic criteria, if applicable	8-9
Data sources/ measurement	8*	For each variable of interest, give sources of data and details of methods of assessment (measurement). Describe comparability of assessment methods if there is more than one group	*8-9*
Bias	9	Describe any efforts to address potential sources of bias	
Study size	10	Explain how the study size was arrived at	N/A
Quantitative variables	11	Explain how quantitative variables were handled in the analyses. If applicable, describe which groupings were chosen and why	8-9
Statistical methods	12	(*a*) Describe all statistical methods, including those used to control for confounding	10-11
		(*b*) Describe any methods used to examine subgroups and interactions	10-11
		(*c*) Explain how missing data were addressed	N/A
		(*d*) *Cohort study*—If applicable, explain how loss to follow-up was addressed<br>*Case-control study*—If applicable, explain how matching of cases and controls was addressed	

For peer review only

		Cross-sectional study—If applicable, describe analytical methods taking account of sampling strategy	
		(*e*) Describe any sensitivity analyses	9
**Results**			
Participants	13*	(a) Report numbers of individuals at each stage of study—eg numbers potentially eligible, examined for eligibility, confirmed eligible, included in the study, completing follow-up, and analysed	
		(b) Give reasons for non-participation at each stage	
		(c) Consider use of a flow diagram	
Descriptive data	14*	(a) Give characteristics of study participants (eg demographic, clinical, social) and information on exposures and potential confounders	12
		(b) Indicate number of participants with missing data for each variable of interest	N/A
		(c) *Cohort study*—Summarise follow-up time (eg, average and total amount)	
Outcome data	15*	*Cohort study*—Report numbers of outcome events or summary measures over time	15
		Case-control study—Report numbers in each exposure category, or summary measures of exposure	
		Cross-sectional study—Report numbers of outcome events or summary measures	
Main results	16	(*a*) Give unadjusted estimates and, if applicable, confounder-adjusted estimates and their precision (eg, 95% confidence interval). Make clear which confounders were adjusted for and why they were included	16
		(*b*) Report category boundaries when continuous variables were categorized	
		(*c*) If relevant, consider translating estimates of relative risk into absolute risk for a meaningful time period	
Other analyses	17	Report other analyses done—eg analyses of subgroups and interactions, and sensitivity analyses	
**Discussion**			
Key results	18	Summarise key results with reference to study objectives	19
Limitations	19	Discuss limitations of the study, taking into account sources of potential bias or imprecision. Discuss both direction and magnitude of any potential bias	21
Interpretation	20	Give a cautious overall interpretation of results considering objectives, limitations, multiplicity of analyses, results from similar studies, and other relevant evidence	22
Generalisability	21	Discuss the generalisability (external validity) of the study results	21
**Other information**			
Funding	22	Give the source of funding and the role of the funders for the present study and, if applicable, for the original study on which the present article is based	23

*Give information separately for cases and controls in case-control studies and, if applicable, for exposed and unexposed groups in cohort and cross-sectional studies.

Note: An Explanation and Elaboration article discusses each checklist item and gives methodological background and published examples of transparent reporting. The STROBE checklist is best used in conjunction with this article (freely available on the Web sites of PLoS Medicine at http://www.plosmedicine.org/, Annals of Internal Medicine at http://www.annals.org/, and Epidemiology at http://www.epidem.com/). Information on the STROBE Initiative is available at www.strobe-statement.org.

For peer review only

BMJ Open

POTENTIALLY AVOIDABLE CAUSES OF HOSPITALISATION IN PEOPLE WITH DEMENTIA: CONTEMPORANEOUS ASSOCIATIONS BY STAGE OF DEMENTIA IN A SOUTH LONDON CLINICAL COHORT

Journal:	*BMJ Open*
Manuscript ID	bmjopen-2021-055447.R1
Article Type:	Original research
Date Submitted by the Author:	19-Jan-2022
Complete List of Authors:	Gungabissoon, Usha; GSK, Epidemiology (Value Evidence and Outcomes); King's College London Institute of Psychiatry Psychology and Neuroscience, Psychological Medicine<br>Perera, Gayan; King's College London Institute of Psychiatry Psychology and Neuroscience, Psychological Medicine<br>Galwey, Nicholas; GSK, Research Statistics<br>Stewart, Robert; King's College London Institute of Psychiatry Psychology and Neuroscience, Psychological Medicine; South London and Maudsley NHS Foundation Trust
**Primary Subject Heading**:	Epidemiology
Secondary Subject Heading:	Geriatric medicine, Health services research, Mental health
Keywords:	Dementia < NEUROLOGY, EPIDEMIOLOGY, Old age psychiatry < PSYCHIATRY

SCHOLARONE™
Manuscripts

I, the Submitting Author has the right to grant and does grant on behalf of all authors of the Work (as defined in the below author licence), an exclusive licence and/or a non-exclusive licence for contributions from authors who are: i) UK Crown employees; ii) where BMJ has agreed a CC-BY licence shall apply, and/or iii) in accordance with the terms applicable for US Federal Government officers or employees acting as part of their official duties; on a worldwide, perpetual, irrevocable, royalty-free basis to BMJ Publishing Group Ltd ("BMJ") its licensees and where the relevant Journal is co-owned by BMJ to the co-owners of the Journal, to publish the Work in this journal and any other BMJ products and to exploit all rights, as set out in our licence.

The Submitting Author accepts and understands that any supply made under these terms is made by BMJ to the Submitting Author unless you are acting as an employee on behalf of your employer or a postgraduate student of an affiliated institution which is paying any applicable article publishing charge ("APC") for Open Access articles. Where the Submitting Author wishes to make the Work available on an Open Access basis (and intends to pay the relevant APC), the terms of reuse of such Open Access shall be governed by a Creative Commons licence – details of these licences and which Creative Commons licence will apply to this Work are set out in our licence referred to above.

Other than as permitted in any relevant BMJ Author's Self Archiving Policies, I confirm this Work has not been accepted for publication elsewhere, is not being considered for publication elsewhere and does not duplicate material already published. I confirm all authors consent to publication of this Work and authorise the granting of this licence.

For peer review only

WORD COUNT: 4100

TITLE: POTENTIALLY AVOIDABLE CAUSES OF HOSPITALISATION IN PEOPLE WITH DEMENTIA: CONTEMPORANEOUS ASSOCIATIONS BY STAGE OF DEMENTIA IN A SOUTH LONDON CLINICAL COHORT

Authors:

Usha Gungabissoon[1,2], M.Sc.; Gayan Perera[1], Ph.D.; Nicholas Galwey[3], Ph.D.; Robert Stewart[1,4], M.D., FRCPsych

[1]Institute of Psychiatry, Psychology and Neuroscience (IOPPN), King's College London, London, United Kingdom (UK)

[2]Epidemiology, Value, Evidence and Outcomes, Global Medical, GlaxoSmithKline (GSK) R&D, London, UK

[3]Research Statistics, GSK R&D, London, UK

[4]South London and Maudsley NHS Foundation Trust, London, UK

For peer review only - http://bmjopen.bmj.com/site/about/guidelines.xhtml

Corresponding author: Usha Gungabissoon

Department of Psychological Medicine (PO84), Institute of Psychiatry, Psychology and Neuroscience, King's College London. De Crespigny Park, London SE5 8AF

Email: usha.2.gungabissoon@gsk.com

Phone: +442080476878

For peer review only

ABSTRACT (word count 299/300)

Objectives

To estimate the frequency of all-cause hospitalisations and those related to ambulatory care sensitive conditions (ACSCs); a proxy for potentially avoidable hospitalisations amongst individuals with dementia, and to investigate differences by stage of dementia based on recorded cognitive function.

Setting

Data from a large London dementia care clinical case register, linked to a national hospitalisation database.

Participants

Individuals aged ≥65 years with a confirmed dementia diagnosis with recorded cognitive function.

Outcome measures

Acute general hospital admissions were evaluated within 6 months of a randomly selected cognitive function score in patients with a clinical diagnosis of dementia. To evaluate associations between ACSC-related hospital admissions (overall and individual ACSCs) and stage of dementia, an ordinal regression was performed, modelling stage of dementia as the dependant variable (to facilitate efficient model selection, with no implication concerning the direction of causality).

Results

Of 5,294 people with dementia, 2,993 (56.5%) had at least one hospitalisation during a 12-month period of evaluation, and 1,192 (22.5%) had an ACSC-related admission. Proportions with an all-cause or ACSC-related hospitalisation were greater in the groups with more advanced dementia (all-cause 53.9%, 57.1% and 60.9%, p 0.002; ACSC-related 19.5%, 24.0% 25.3%, p <0.0001in the mild, moderate and severe groups respectively). An ACSC-related admission was associated with 1.3-fold increased odds of more severe dementia after adjusting for demographic factors. Concerning admissions for individual ACSCs, the most common ACSC was UTI/pyelonephritis (9.8% of hospitalised patients) followed by pneumonia (7.1%);

For peer review only - http://bmjopen.bmj.com/site/about/guidelines.xhtml

in an adjusted model, these were each associated with 1.4-fold increased odds of more severe dementia.

Conclusions

Potentially avoidable hospitalisations were common in people with dementia, particularly in those with greater cognitive impairment. Our results call for greater attention to the extent of cognitive status impairment, and not just dementia diagnosis, when evaluating measures to reduce the risk of potentially avoidable hospitalisations.

Key words: dementia, epidemiology, healthcare utilisation, avoidable hospitalisation, ambulatory care sensitive

For peer review only

For peer review only - http://bmjopen.bmj.com/site/about/guidelines.xhtml

Strengths and limitations of this study

- Through linkage of a catchment specialist healthcare case register to a national hospitalisation database, our study benefited from near-complete outcome ascertainment enabling us to evaluate potentially avoidable causes of hospitalisation in a large sample of clinically confirmed cases of dementia.
- Severity of dementia was ascertained from routinely administered cognitive assessments, making use of natural language processing algorithms with established validity.
- Reasons for hospitalisation were derived from the discharge diagnoses recorded for last episode of the hospital admission, meaning it was not possible to distinguish complications arising during the hospital stay from reasons for the initial admission.
- Data were obtained from single service provider in an urban catchment area, which may limit the generalisability of our findings to other settings.
- The analyses did not account for comorbidities, use of medications or living arrangement (such as home versus care home); furthermore, dementia stage and hospitalisations were measured contemporaneously, meaning that conclusions about causation should not be drawn.

For peer review only

INTRODUCTION

Dementia represents a significant challenge for health services. For example, in the UK National Health Service (NHS) an estimated 25% of acute beds are occupied by people with dementia[1] and recent analyses have shown a 35% increase in the number of emergency hospital admissions for people with dementia in England over the past five years.[2] In 2016, the global economic costs caused by dementia approached $1 trillion, with the highest economic burden seen in Europe and North America.[3] Major contributing factors to costs include disease severity and higher rates of hospitalisation in people with dementia relative to older people without dementia.[4]

Whilst some hospitalisations are necessary and expected, others are potentially avoidable. Ambulatory care sensitive conditions (ACSCs) are conceptualised as chronic and acute health conditions such as heart failure, pneumonia, pyelonephritis and complications of diabetes, for which effective primary and community care may avoid the need for hospital admission by preventing the onset of these diseases, controlling the acute episodic illness, or managing the chronic condition more effectively.[5 6] ACSC-related admissions, for example, are used to assess how the NHS is performing in England.[7]

For peer review only - http://bmjopen.bmj.com/site/about/guidelines.xhtml

Preventing hospitalisation is of particular importance in people with dementia; hospitalisation is associated with poorer outcomes including prolonged hospital stays and increased risk of complications relative to people without dementia.[8-11] Further, hospitalisation itself can trigger distress, or confusion which can contribute to a decline in functioning creating new care challenges, or can intensify the existing burden of care.[12]

Even at early stages of cognitive impairment, a person's ability to make health care decisions can be altered, [13] including changes in health-seeking behaviours and management of existing conditions [14]. This could explain why the age-adjusted risk of potentially avoidable hospitalisations is greater in people with dementia than those without. [9 10] There is also some evidence suggesting that the risk of potentially avoidable hospitalisations is greater in people with later-stage dementia.[8 15] However, previous studies have been small, used non-representative convenience samples or relatively crude methods to evaluate cognitive deficits. Understanding which potentially avoidable causes of hospitalisation predominate at later stages of dementia can help to tailor healthcare needs in this population in order to prevent the need for hospitalisation.

For peer review only - http://bmjopen.bmj.com/site/about/guidelines.xhtml

In the present study, we used data drawn from a mental healthcare case register linked to national hospitalisation episodes, to describe ACSC-related hospitalisations, a proxy for potentially avoidable hospitalisations, amongst a large sample of patients with dementia. Specifically, we sought first to investigate whether ACSC-related hospital admission was associated with more advanced stages of dementia, and second to explore whether hospitalisations for specific ACSCs were associated with more advanced dementia stage.

For peer review only

For peer review only - http://bmjopen.bmj.com/site/about/guidelines.xhtml

METHODS

Study design

This study investigated acute general hospital admissions around the time of a given cognitive function score (index date) in a sample of patients with a clinical diagnosis of dementia using linked clinical datasets.

Data sources and sample

The Clinical Record Interactive Search (CRIS) data resource was used to identify dementia cases. CRIS provides research access to anonymised electronic health records (EHRs) within the South London and Maudsley NHS Foundation Trust (SLaM). The trust is a provider of mental healthcare, dementia assessment and management, for a south London catchment of >1.2 million residents in Lambeth, Lewisham, Croydon and Southwark; EHRs have been implemented across all SLaM services since 2006.[16].

Routine diagnoses are recorded using the International Classification of Diseases 10th edition (ICD-10) coding system, and are supplemented in CRIS by a natural language processing (NLP) to abstract measures of cognitive impairment.[16 17] Hospital outcomes (hospital admissions and their causes) for patients with dementia were obtained from the a linkage with

For peer review only - http://bmjopen.bmj.com/site/about/guidelines.xhtml
For peer review only

the Hospital Episode Statistics (HES) database. HES contains details of all inpatient admissions at NHS hospitals in England.[18] Discharge diagnoses are also recorded as ICD-10 codes and are available for each hospitalisation episode. HES data up to 31st March 2013 were available at the time of analysis.

For peer review only

Study population

Patients with a diagnosis of dementia first recorded in SLaM between 1 January 2006 and 30th September 2012, and aged ≥65 years at diagnosis, were identified on CRIS. Dementia was defined on the presence of F00*, F01*, F02*or F03* ICD diagnosis codes recorded up to the sixth position in the structured data. Eligible patients were those who had at least one cognitive assessment (from structured data or free text using NLP) recorded: either a Mini Mental State Examination score (MMSE) or a cognitive impairment score from the Health of the Nation Outcome Scales (HoNOS). HoNOS is a clinician-rated instrument usually completed on a structured field within the EHR at first assessment, which contains subscales rated 0 (no

For peer review only - http://bmjopen.bmj.com/site/about/guidelines.xhtml

problem) to 4 (severe/very severe problem); its cognitive scale correlates with MMSE measurement.[19] To exclude patients with potential mild cognitive impairment rather than dementia, the sample was further restricted to those with an MMSE score of <28 or HoNOS cognitive impairment score >1. In addition, we excluded patients who received their first dementia diagnosis from an acute hospital liaison service; these reflected cases where the recording of a dementia diagnosis might have been precipitated by a hospitalisation and by definition be a group with significant hospitalised physical disorders, therefore potentially biasing any associations between dementia and comorbidity.

For each eligible patient, we randomly selected a single measure of cognition recorded in the patient's clinical record using a random number generator in Microsoft Excel; the date for the measure was assigned as the index date. This process was applied hierarchically: if several MMSE scores were recorded over time for a given patient, then one was randomly selected; for those individuals without an MMSE measurement, a random Health of the Nation Outcome Scales (HoNOS) cognitive impairment subscale was selected in the same manner. The randomly selected score for each patient was used to categorise the stage of dementia into mild, moderate or severe on the date of the cognitive assessment as described below.

For peer review only - http://bmjopen.bmj.com/site/about/guidelines.xhtml

Measurements

The following demographic information was obtained from CRIS: age at diagnosis of dementia, sex, ethnicity (White-European, other, not specified), dementia diagnostic group (Alzheimer's, vascular, mixed or other) and Index of Multiple Deprivation (IMD) score, a widely used measure of relative neighbourhood deprivation.[20] IMD is derived from Census data and calculated for clusters of adjacent unit postcodes in England known as Lower layer Super Output Areas (LSOA), standard geographic units with around 1500 residents; a higher IMD score indicates a more deprived area.

To classify dementia stage at the index date, we used cut-offs similar to those used by others[21], defining MMSE scores of >20, 10-20, and <10, or a HoNOS cognitive impairment subscale score of 2, 3, and 4, as representing mild, moderate, or severe dementia, respectively.

Acute general hospital inpatient admissions were obtained for the 6-month period either side of the index date, i.e., over a 12-month period. Hospitalisations were defined from HES episodes, combining contiguous episodes (i.e., where start and end dates were on the same day); in the context of this analysis, an admission, or spell, is defined as a continuous period

For peer review only - http://bmjopen.bmj.com/site/about/guidelines.xhtml

spent as a patient in hospital. Four-digit ICD-10 discharge diagnoses recorded during the last episode of each hospital admission were obtained (up to twenty diagnoses may be recorded as part of a hospital episode).

Ambulatory care sensitive conditions (ACSC) were identified based on the presence of specific ICD-10 codes and their position in the record, using a modification of the approach used by Bardsley *et al* and the UK Health and Social Care Information Centre; for example, angina is based on the presence of specific ICD-10 codes in the primary position, whereas pneumonia is defined based on the presence of a relevant code in any position.[5 6] Deviations from the published approach comprised the following: i) for the UTI category, we deemed cystitis to be relevant and therefore included relevant codes in this group; ii) we removed O15 (eclampsia) from the convulsions and epilepsy category as used by Bardsley. Each ACSC was specified individually as a binary variable to flag the presence of a hospitalisation with that specific condition. Given that diagnoses may not be recorded in the order of clinical importance in the real-world setting, as a sensitivity analysis, we relaxed the requirement for conditions which were based on a diagnosis in the primary position only, to include diagnoses in either of the

For peer review only

For peer review only - http://bmjopen.bmj.com/site/about/guidelines.xhtml

first two positions. **Supplementary table 1** lists the ACSCs, the ICD codes and the position of the ICD code.

Additional covariates included binary variables to indicate an all-cause or any ACSC-related hospitalisation during the 12-month observation period. Time since first recorded dementia diagnosis (a proxy for disease duration) in SLaM at index date was also obtained.

Statistical analyses

Microsoft Excel was used for data management, and Stata 15·0 was used for statistical analyses. Patient characteristics were described by stage of dementia; normally distributed variables were presented as mean ± standard deviation (SD) and non-normally distributed variables as median and upper and lower quartiles. For the bivariate analysis, comparison of continuous variables between dementia stages (mild, moderate, severe) was evaluated using ANOVA, with the exception of number of inpatient admissions and time since presentation at SLaM which were evaluated by the Kruskal-Wallis test due to their non-normal distribution. For the relationship between each categorical variable and stage of dementia, a chi-squared test of association was performed, and percentage distributions presented.

For peer review only

For peer review only - http://bmjopen.bmj.com/site/about/guidelines.xhtml

To evaluate the association between any ACSC-related hospitalisation and contemporaneously measured stage of dementia, unadjusted, age-sex-adjusted and fully adjusted odds ratios (OR) were obtained from generalised ordinal logistic regression models,[22] with dementia stage (mild, moderate, severe) as the dependant variable. Including dementia stage as the outcome variable was deemed to be the most efficient approach to evaluate ACSC-related hospitalisations and specific causes of ACSC-related admissions as it allowed us to use the efficient, flexible approach of stepwise model selection, to evaluate the importance of the relationship with other variables. Because our aim was to assess co-temporal associations between stage and hospitalisations rather than causality, we considered that whether dementia stage was modelled as a response, or as an explanatory variable, was not critical.

To investigate whether specific causes of ACSC-related admissions were associated with dementia stage, a backward stepwise model selection was used to build a multivariable generalised ordinal logistic regression model, with dementia stage as the dependant variable. We used the `gologit2` STATA command with the `autofit` option to selectively relax the model, removing the proportional odds assumption for any variable for which this assumption was violated.[22] Sex, time from first dementia record at index date (years) and age-group at

For peer review only - http://bmjopen.bmj.com/site/about/guidelines.xhtml

index date were deemed clinically relevant and were therefore forced into the model (i.e., retained regardless of their p-value). Variables with p<0.1 in the bivariate analysis were initially included in the stepwise model selection. During stepwise selection, variables with p <0.2 were retained in the model, and following exclusion of any variable, the variables already excluded were considered for re-inclusion. Collinearity was evaluated based on the Variance Inflation Factor (VIF) and tolerance for each variable; covariates with a VIF greater than 2.5 were removed one at a time, starting with variables with the largest VIF, and the impact on remaining covariates was assessed until no remaining variable had a VIF >2.5. Further, interactions between age-group and sex, and between age-group and dementia diagnostic group, were assessed for possible inclusion, based on the Akaike Information Criterion (AIC).

Patient and Public Involvement

Patients or the public were not involved in the design, or conduct, or reporting, or dissemination plans of our research.

For peer review only

RESULTS

In total, 5,294 patients with dementia met the study criteria and were included in the analysis sample. Almost two-thirds of the sample were female (N=3,393, 64.1%) and the mean age at the date of randomly chosen cognitive assessment was 82.5 years (SD 6.8). The proportions of patients with mild, moderate and severe dementia at these index dates were 37.3%, 46.4% and 16.1% respectively. Descriptive characteristics by stage of dementia are presented in **Table 1.**

Table 1: Descriptive analysis by dementia severity (N=5,294)

		Mild (N=1,979)	Moderate (N=2,461)	Severe (N=854)	All (N=5,294)	Test statistic [d.f] p value
**Age at index date N (%)**	65-74y	326 (16.5)	292 (11.9)	116 (13.6)	734 (13.9)	χ^2 [4] = 23.35 p <0.0001
	75-84y	892 (45.1)	1107 (45.0)	397 (46.5)	2396 (45.3)	
	85+	761 (38.5)	1062 (43.2)	341 (39.9)	2164 (40.9)	
**Mean age at index date (SD)**		81.9 (6.9)	82.9 (6.8)	82.4 (6.9)	82.5 (6.8)	F [2, 5291] = 11.96 p <0.0001
**Sex**	Female	1233 (62.3)	1615 (65.6)	545 (63.8)	3393 (64.1)	χ^2 [2]=5.29 p 0.071
	Male	746 (37.7)	846 (34.4)	309 (36.2)	1901 (35.9)	
**Ethnic group**	White-European	1614 (81.6)	1915 (77.8)	655 (76.7)	4184 (79.0)	χ^2 [4] = 9.54 p 0.008
	Other	337 (17.0)	502 (20.4)	172 (20.1)	1011 (19.1)	
	Not Specified	28 (1.4)	44 (1.8)	27 (3.2)	99 (1.9)	

For peer review only - http://bmjopen.bmj.com/site/about/guidelines.xhtml

**Deprivation score - mean (SD)**		26.02 (11.4)	27.46 (11.0)	28.1 (11.0)	27.02 (11.2)	F [2, 5260] = 13.75 p <0.0001
**Time (years) since first presentation at SLaM – median (IQR)**		0.31 (0.03 ,0.91)	0.31 (0.02 ,1.21)	0.31 (0.02 ,1.28)	0.31 (0.02 ,1.06)	H (2) = 1.04 p 0.596
**Dementia diagnostic group**	Alzheimer's	852 (43.1)	931 (37.8)	336 (39.34)	2119 (40.03)	χ^2 [6] = 25.81 p <0.0001
	Mixed	206 (10.4)	278 (11.3)	74 (8.7)	558 (10.5)	
	Vascular	385 (19.5)	602 (24.5)	197 (23.1)	1184 (22.4)	
	Other	536 (27.1)	650 (26.4)	247 (28.9)	1433 (27.1)	.

Percentages presented in parentheses unless otherwise specified

Test statistics: χ^2 Chi square test of association, F: One-way ANOVA, H: Kruskal-Wallis test.

d.f: degrees of freedom

All-cause and ACSC-related hospitalisations

Overall, 56.5% (N=2,993 of 5,294) of individuals analysed had at least one inpatient admission in the 6-month period either side of the index date, and this percentage was greater with more advanced dementia: 53.9% of patients with mild, 57.1% with moderate and 60.9% with severe dementia (χ^2 (d.f. 2) =12.47, p = 0.002) (**Table 2**).

Within the cohort, 22.5% had at least one ACSC-related hospitalisation (N=1,192/5,294) which accounted for almost 40% of patients with any hospital admission (N=1,192/2,993). ACSC-related hospitalisations were observed in 36.2%, 42.0% and 41.5% of hospitalised patients with mild, moderate and severe dementia respectively (χ^2 (d.f. 2) =9.25 p = 0.01). The most

For peer review only

common ACSC was UTI/pyelonephritis (9.8% of hospitalised patients) followed by pneumonia (7.1%), both of which tended to be more frequent with more severe dementia (p <0.0001 for both conditions). The mean number of ACSC admissions amongst hospitalised patients was highest in those with moderate (0.67, SD 1.04) compared to mild or severe dementia (0.57, SD 1.01 and 0.58, SD 0.85, F [2, 2990] = 3.33, $p = 0.036$).

For peer review only

For peer review only - http://bmjopen.bmj.com/site/about/guidelines.xhtml

Table 2: Hospital admissions by severity of dementia (N=5,294)

	Mild (N=1979)	Moderate (N=2461)	Severe (N=854)	All (N=5294)	Test statistic [d.f] p value
Inpatient admission +/- 6 months of index date, N (%)	1067 (53.9)	1406 (57.1)	520 (60.9)	2993 (56.5)	χ^2 [2] = 12.47 p 0.002
Number of all cause admissions per patient – Median (lower and upper quartile)*	2 (1,3)	2 (1,3)	2 (1,3)	2 (1,3)	H [2] = 3.42 p 0.181
Ambulatory care sensitive conditions□ N (% of all patients)					
Angina	27 (1.4)	28 (1.1)	5 (0.6)	60 (1.1)	χ^2 [2] = 3.2 p 0.199
Asthma	2 (0.1)	2 (0.1)	1 (0.1)	5 (0.1)	χ^2[2] = 0.10 p 0.951
Cellulitis	19 (1.0)	27 (1.1)	9 (1.1)	55 (1.0)	χ^2 [2] = 0.20 p 0.904
Congestive heart failure	28 (1.4)	38 (1.5)	10 (1.2)	76 (1.4)	χ^2 [2] = 0.63 p 0.729
Convulsion/epilepsy	16 (0.8)	23 (0.9)	12 (1.4)	51 (1.0)	χ^2 [2] = 2.26 p 0.322
COPD	28 (1.4)	40 (1.6)	8 (0.9)	76 (1.4)	χ^2 [2] = 2.13 p 0.344
Dehydration	8 (0.4)	20 (0.8)	7 (0.8)	35 (0.7)	χ^2[2] = 3.18 p 0.204
Dental	9 (0.5)	10 (0.4)	1 (0.1)	20 (0.4)	χ^2 [2] = 1.91 p 0.385
Diabetes complications	30 (1.5)	48 (2.0)	11 (1.3)	89 (1.7)	χ^2 [2] = 2.205 p 0.332
ENT infections	5 (0.3)	2 (0.1)	1 (0.1)	8 (0.2)	χ^2 [2] = 2.21 p 0.331
Gastroenteritis	21 (1.1)	20 (0.8)	3 (0.4)	44 (0.8)	χ^2 [2] = 3.67 p 0.16
Hypertension	3 (0.2)	3 (0.1)	-	6 (0.1)	χ^2 [2] = 1.24 p 0.538
Iron deficiency anaemia	6 (0.3)	15 (0.6)	2 (0.2)	23 (0.4)	χ^2 [2] = 3.32 p 0.19
Other vaccine preventable	2 (0.1)	-	-	2 (0.04)	χ^2 [2] = 3.35 p 0.187

For peer review only

For peer review only - http://bmjopen.bmj.com/site/about/guidelines.xhtml

**Perforated/bleeding ulcer**	-	3 (0.1)	3 (0.4)	6 (0.1)	χ^2 [2] = 6.53 p 0.038
**Pneumonia**	103 (5.2)	201 (8.2)	70 (8.2)	374 (7.1)	χ^2 [2] = 16.653 p <0.0001
**UTI pyelonephritis**	155 (7.8)	255 (10.4)	111 (13.0)	521 (9.8)	χ^2 [2] = 19.34 p <0.0001
**Any ACSC admission N (%)**					
**overall**	386 (19.5)	590 (24.0)	216 (25.2)	1192 (22.5)	χ^2 [2] = 17.06 p <0.0001
**amongst those with an inpatient admission**	386 (36.2)	590 (42.0)	216 (41.5)	1192 (39.8)	χ^2 [2] = 9.25 p 0.010
**Mean number of ACSC admissions per patient (SD)**					
**amongst those with an inpatient admission**	0.6 (1.0)	0.7 (1.0)	0.6 (0.9)	0.6 (1.0)	F [2, 2990] = 3.33 p 0.036

□ No admissions meeting the criteria for flu or gangrene identified

***** amongst hospitalised

Test statistics: χ^2 Chi square test, F: One-way ANOVA, H: Kruskal-Wallis test

d.f: degrees of freedom

For peer review only

For peer review only - http://bmjopen.bmj.com/site/about/guidelines.xhtml

Associations between any ACSC-related hospitalisation and dementia stage

Any ACSC-related hospitalisation was significantly associated with more advanced dementia, i.e., an OR significantly greater than 1 for moderate/severe vs. mild, or severe vs. mild/moderate, in the unadjusted model (unadjusted OR 1.28, 95% CI 1.14, 1.45). The association remained significant in the fully adjusted model which accounted for age, sex, time since first presentation on SLaM, ethnic-group and neighbourhood deprivation (OR 1.27, 95% CI 1.12, 1.44 for both moderate/severe vs. mild and severe vs. mild/moderate) (**Table 3**).

For peer review only

Table 3: Unadjusted and adjusted ORs and 95% CIs for any ACSC-related admission by stage of dementia

	**Severe/moderate vs. mild <u>or</u> Severe vs. mild/moderate□**<br><br>**OR (95% CI)**
**Unadjusted**	1.28 (1.14, 1.45)
**Age, sex adjusted**	1.27 (1.13, 1.44)
**Fully adjusted***	1.27 (1.12, 1.44)

*Age-group, sex, time since first presentation on SLaM, ethnicity and IMD

□From a generalised ordinal logistic regression model: the same odds ratio applies to both comparisons as the proportional odds assumption is not violated.

Exploratory analysis: associations between specific ACSC-related hospitalisations and dementia stage

Variables included in the multivariable model after stepwise selection are summarised in **Table 4**. The goodness of fit of the final model, as measured by the likelihood ratio chi-square test, demonstrated that the model as a whole fitted significantly better than the null model (i.e., a model with no predictors; LR Chi square 144.6, p <0.0001). The output from the stepwise-selected regression model is presented in **Table 4**. The proportional odds assumption was violated for age-group, and dementia diagnostic group, meaning that the effect of these explanatory variables was not the same at the two dementia-severity boundaries.

The model indicated that, conditional on other explanatory variables, a greater time since first presentation at SLaM, higher IMD score (i.e., residence in a more deprived area) and "other" ethnicity were associated with greater odds of more severe dementia. Greater age was associated with greater odds of moderate/severe dementia relative to mild, but not of severe dementia relative to mild/moderate.

Considering specific conditions, perforated/bleeding ulcer, UTI/pyelonephritis and pneumonia hospitalisations were all significantly associated with greater odds of more advanced dementia, i.e., moderate/severe relative to mild or severe relative to mild/moderate. Although a strong

For peer review only - http://bmjopen.bmj.com/site/about/guidelines.xhtml
For peer review only

association between perforated/bleeding ulcer and dementia stage (OR 7.01 *p* 0.01) was observed, it is important to note that in terms of absolute frequency this condition was uncommon, being recorded in only 6 individuals. UTI/pyelonephritis and pneumonia were also associated with higher odds of more severe dementia (OR 1.38 p <0.0001, OR 1.38 p 0.002 respectively); this was consistent across dementia severity categories, i.e., the ORs and p-values apply to both cut-points. Gastroenteritis-related admissions had an inverse relationship with dementia-stage, namely that admission was associated with a lower odds of advanced dementia stages: however, this was of borderline significance (OR 0.57 p 0.06).

Table 4: Results from stepwise-selected partial generalised ordinal logistic regression model for severity of dementia (N=5,294)*

Explanatory variable	Moderate/severe vs. mild dementia		Severe vs. mild/moderate Dementia	
	OR (95% CI)	p	OR (95% CI)	p
Sex (male reference)	1.07 (0.96, 1.2)	0.203	1.07 (0.96, 1.2)	0.203
Time since first presentation at SLaM	1.13 (1.08, 1.19)	<0.0001	1.13 (1.08, 1.19)	<0.0001
Age group at index date (65-74 years reference)				
	1.36 (1.15, 1.62)	<0.0001	1.04 (0.82, 1.31)	0.763
	1.53 (1.28, 1.83)	<0.0001	1.01 (0.79, 1.28)	0.949
Deprivation score	1.01 (1.01, 1.02)	<0.0001	1.01 (1.01, 1.02)	<0.0001
Dementia diagnostic group (AD reference)				

For peer review only - http://bmjopen.bmj.com/site/about/guidelines.xhtml

**Vascular**	1.34 (1.15, 1.56)	<0.0001	1.02 (0.84, 1.24)	0.825
**Mixed**	1.16 (0.95, 1.4)	0.145	0.8 (0.61, 1.05)	0.114
**Other**	1.12 (0.98, 1.27)	0.099	1.12 (0.98, 1.27)	0.099
Ethnic group (European reference)				
**Other**	1.2 (1.05, 1.37)	0.009	1.2 (1.05, 1.37)	0.009
**Pneumonia**	1.38 (1.13, 1.7)	0.002	1.38 (1.13, 1.7)	0.002
**Perforated/bleeding ulcer**	7.01 (1.56, 31.57)	0.011	7.01 (1.56, 31.57)	0.011
**Dehydration**	1.71 (0.92, 3.17)	0.09	1.71 (0.92, 3.17)	0.09
**Gastroenteritis**	0.57 (0.32, 1.02)	0.059	0.57 (0.32, 1.02)	0.059
**UTI pyelonephritis**	1.38 (1.15, 1.65)	<0.0001	1.38 (1.15, 1.65)	<0.0001

* Proportional odds assumption violated for age and dementia diagnostic group. Note that where it is not violated, the same odds ratio applies to both comparisons.

For a continuous or an ordinal variable, the odds ratio is calculated relative to a one-unit change in the variable. For ACSCs, the reference category is absence of the condition recorded.

ACSC: ambulatory care sensitive condition

Goodness of fit: Likelihood ratio test - LR Chi square 144.6, $p<0.0001$

Sensitivity analysis (expanded ACSC definition)

In addition to perforated/bleeding ulcer (OR 6.92), pneumonia (OR 1.38) and UTI pyelonephritis (OR 1.49), in the sensitivity analysis where the ACSC definition was relaxed, convulsion/epilepsy (OR 1.58) was also found to be significantly associated with more severe dementia (**Supplementary table 2**).

For peer review only - http://bmjopen.bmj.com/site/about/guidelines.xhtml

For peer review only

For peer review only - http://bmjopen.bmj.com/site/about/guidelines.xhtml

DISCUSSION

We evaluated all-cause and potentially avoidable-causes of hospitalisation around the times of randomly selected cognitive function assessments in people with clinically diagnosed dementia, modelling mild vs. moderate vs. severe dementia at the index dates in question. Over half of our sample had a hospitalisation of any kind during a 12-month period of evaluation, and almost a quarter had an ACSC-related hospital admission, which accounted for nearly 40% of hospitalised patients. The proportions of patients with at least one hospitalisation overall, or with one ACSC-related hospitalisation, were greater in the groups with more advanced dementia. The odds of being in a more advanced stage was almost 1.3-fold higher for those with an ACSC-related admission after adjusting for demographics, time from first dementia record at index date (a proxy for disease duration) and neighbourhood deprivation.

For peer review only

Around 6% of community-dwelling individuals with dementia in a US Veterans Health Administration database had at least one ACSC admission during a 12-month period; however, Medicare facility-use was not included, and hospitalisations may thus have been underestimated.[23] In another study, an average of 28% of Medicare beneficiaries per year aged ≥65 years with dementia participating in the Washington Heights-Inwood Columbia Aging

For peer review only - http://bmjopen.bmj.com/site/about/guidelines.xhtml

Project (WHICAP), had an ACSC-related admission,[8] which was more consistent with our own findings.

After adjusting for a range of clinical and demographic variables, we found that an ACSC-related admission remained associated with more advanced dementia. Hospitalisations with UTI/pyelonephritis, pneumonia and perforated/bleeding ulcer recorded as part of an admission were associated with more advanced dementia in our study. Although these have been reported as common causes of admission in people with dementia relative to those without dementia,[24-26] there is sparse literature on relationships with dementia severity. A study of Medicare beneficiaries with dementia found that that worse cognitive status was associated with higher risks of hospitalisations for diabetes, pneumonia, hypertension, and dehydration, and that worse functional status was associated with higher risks of hospitalisation for diabetes and UTI, marginally higher for dehydration, but lower for hypertension.[8]

UTI/pyelonephritis was recorded in 17.4% of hospitalised patients with dementia in our sample, and its presence was associated with more severe dementia. UTIs in more severe dementia may reflect worse immune function,[27] problems with mobility associated with urinary

For peer review only - http://bmjopen.bmj.com/site/about/guidelines.xhtml

incontinence and subsequent infection,[28] and inactivity or dehydration.[29] Consistently, we also observed a suggestion of association with dehydration in more severe dementia, although this was not significant (p = 0.09). The presence of acute urinary symptoms and laboratory evidence (such as a positive urine culture) is required to make a formal UTI diagnosis. Difficulties collecting a urine specimen, an inability to accurately report symptoms, together with the high prevalence of asymptomatic bacteriuria in people with dementia may lead to misdiagnosis, particularly in those whose dementia is severe.[30 31] Further, non-specific symptoms such as new onset or worsening of confusion is a common reason suspecting a UTI in elderly patients and may also lead to over-diagnosis in this population.[32] These factors may confound our findings.

Hospitalised pneumonia was common in our sample (12.5% of hospitalised patients with dementia) and was associated with more advanced dementia, as has been described by others.[33 34] Uptake of the pneumococcal vaccine in the over-65 age-group is relatively high in the UK (69.5% of the primary care population)[35]: this suggests that pneumonia in our sample may be due to other bacterial or viral causes, or may be occurring as a result of an impaired immune system, dysphagia or urinary incontinence, which are common in, and associated with a higher

For peer review only - http://bmjopen.bmj.com/site/about/guidelines.xhtml

For peer review only

risk of pneumonia in, the elderly.[36 37] Furthermore, it was not possible to determine whether instances of pneumonia identified in our sample were acquired within hospital as part of the inpatient episode, or in the community prior to hospitalisation.

Perforated/bleeding ulcer related admissions were strongly associated with more severe dementia at the index date. A lack of literature on this topic, meant that we were unable to corroborate our findings. Importantly, as only 6 individuals had an admission for this ACSC, limited conclusions can be drawn from our analysis. An understanding of the use of NSAIDs (as a risk factor for GI bleeding) and use of and adherence to proton pump inhibitors which are indicated for the management and/or prevention of GI bleeding by dementia severity would be useful to provide context to our observations.

Lastly, we observed that the mean number of ACSC admissions amongst hospitalised patients was highest in those with moderate compared to mild or severe dementia. It is possible that this observation reflects the increased support that people with more advanced dementia receive.[38] For example, compared to those with moderate dementia, individuals with severe dementia may quality for support (such as home health care) or be more likely to live in assisted

For peer review only

For peer review only - http://bmjopen.bmj.com/site/about/guidelines.xhtml

accommodation or care homes where there is greater support to manage ACSCs on a day-to-day basis. For those residing in the community, caregiver burden has been reported to be associated with an increased risk of hospitalisation.[39 40]

There is an existing body of evidence evaluating various models of care on the risk of hospitalisation including care management, counselling/self-help, enhanced GP services or memory clinics, and physiotherapy or occupational therapy, although none have demonstrated a clear benefit.[41] GP practice factors such as a larger practice population (which may have greater experience with dementia care and/or share patient care between GPs), quality of care, and/or an increasing proportion of the local clinical budget allotted to mental health are reported to be associated with a slight decrease in the rate of avoidable and unavoidable hospital admissions.[42] Others have reported poor continuity of care as a risk factor for hospitalisation.[43] Future care models could include a multi-agency approach including caregiver support.

Strengths and limitations

Our study evaluated hospitalisations in a large sample of clinically diagnosed cases of dementia where cognitive assessment is performed as part of routine clinical care. Linkage of our clinical

For peer review only - http://bmjopen.bmj.com/site/about/guidelines.xhtml

dataset to a national hospitalisation database benefited from near-complete outcome ascertainment, since hospitalisation is free at the point of delivery in the UK context. We also benefited from information allowing differentiation between mild, moderate and severe dementia. Considering limitations, the analysed sample was from a single urban and suburban catchment and included only cases with dementia diagnosed in specialist services; however, the estimated proportion of people with dementia in the SLaM catchment who receive a specialist diagnosis is relatively high at 75.2%.[44] Considering reasons for hospitalisation, these were derived from the discharge diagnoses recorded for last episode of the hospital admission, and it was not possible to distinguish complications that arose during the hospital stay from reasons for the initial admission. Further, scrutiny of a patient's medical history prior to hospitalisation would be required to determine whether a particular ACSC-related admission was truly preventable. Thus, ACSC-related admissions serve as indicators of, rather than definitive measures of, sub-optimal care in the primary-care or community-care settings. Considering residual confounding, this analysis did not account for comorbidity burden or medication use (including polypharmacy) which are potentially associated with hospitalisations and dementia severity,[9 11 23 40 45] nor did it account for prior healthcare utilisation, functional ability,[45] or psychiatric symptoms. Presence of frailty, living

For peer review only

arrangement, available support (family, community and home health support), care-giver burden and GP practice level factors are associated with hospitalisation [40 42] but were not available in this analysis.

Patients with severe dementia were under-represented in our sample and the time since first diagnosis in SLaM in these patients was relatively short despite the extent of cognitive impairment; it is therefore possible that our patients with severe dementia are generally those presenting late in their disease course, and not representative of the general advanced-dementia population. Our analysis evaluated associations between hospitalisation patterns and contemporaneously measured severity of dementia, so it is not appropriate to draw conclusions about causation. In addition, we evaluated hospitalisation outcomes up to 6-months after a cognitive measure and did not attempt to account for survival effects. Mortality increases with severity of dementia[34], and is a competing risk for hospitalisation, although the relatively short timeframe evaluated should mitigate the effects of this.

For peer review only - http://bmjopen.bmj.com/site/about/guidelines.xhtml

Conclusions

Our study adds to a small but increasing body of evidence on the relationship between dementia and potentially avoidable hospitalisations. Despite the clinical and economic burden, we are not aware of any studies in the UK setting that describe potentially avoidable hospitalisations in patients with dementia. We show that potentially avoidable hospitalisations are common in people with dementia, and in particular in those with more advanced dementia; these findings are of importance given that hospitalisation is a significant element in the cost of dementia care and that the number of people with dementia is increasing. Greater attention to the extent of cognitive impairment, and not only the presence of a dementia diagnosis, is needed in order to prevent hospitalisations. However, more work is clearly needed to better understand factors contributing to the high rates of ACSC-related hospitalisation, including the management of ACSCs in the primary care setting. Reducing admissions for ACSCs represents an opportunity to improve both the quality and efficiency of care in healthcare systems.

Ethics statements

Patient consent for publication

Not required.

Ethics approval

For peer review only - http://bmjopen.bmj.com/site/about/guidelines.xhtml

The Oxford Research Ethics Committee C (reference 18/SC/0372) approved CRIS at the Maudsley as a data resource for secondary analysis.

Author contributions

UG was responsible for the design, analysis, interpretation and drafting of the manuscript. GP contributed to the drafting of the manuscript. NG provided statistical input and contributed to the drafting of the manuscript. RS contributed to the design, interpretation and drafting of the manuscript

Funding

This work was supported by NIHR Biomedical Research Centre at the South London and Maudsley NHS Foundation Trust and King's College London. RS is additionally part-funded by an NIHR Senior Investigator Award, by the National Institute for Health Research (NIHR) Applied Research Collaboration South London (NIHR ARC South London) at King's College Hospital NHS Foundation Trust, and by the DATAMIND HDR UK Mental Health Data Hub (MRC grant MR/W014386).

Role of the funding source

The funder of the study had no role in the study design, data collection, data analysis, data interpretation, or writing of the report. UG, GP and RS had access to the anonymised data. The corresponding author had the final responsibility for the decision to submit for publication.

For peer review only - http://bmjopen.bmj.com/site/about/guidelines.xhtml

Declaration of Competing Interest

UG and NG are employees of GSK, hold stock and receive a salary from GSK. RS has received research support in the last 5 years from Janssen, Takeda and GSK.

Acknowledgments

The authors would like to acknowledge Hitesh Shetty for his contributions to the study.

Data sharing

Because of their nature and to comply with their ethical approval, CRIS data are required to remain within the firewall of the South London and Maudsley NHS Foundation Trust (SLaM). Access to the data used for this study can be facilitated by the CRIS Oversight Committee on application and with appropriate SLaM affiliation, details of which can be obtained from cris.administrator@slam.nhs.uk.

For peer review only

For peer review only - http://bmjopen.bmj.com/site/about/guidelines.xhtml

References

1. Dementia Statistics Hub. Dementia Statistics. Hospitals online [Available from: https://www.dementiastatistics.org/statistics/hospitals/ accessed 19/03/2021 2021.
2. Alzheimer's Society. Analysis of NHS England's Hospital Episode Statistics dataset 2012/13 to 2017/18: Alzheimer's Society, 2020.
3. Xu JZ, Y.; Qiu, C.; Cheng, F. Global and regional economic costs of dementia: a systematic review. *The Lancet* 2017;390:S47. doi: 10.1016/S0140-6736(17)33185-9
4. Prince MK, M.; Guerchet, M.; McCrone, P.; Prina, M.; Comas-Herrera, A.; Wittenberg, R.; Adelaja, B.; Hu, B.; King, D.; Rehill, A.; Salimkumar, D.;. Dementia UK Update, 2014.
5. NHS Digital. Enhancing quality of life for people with long-term conditions. In: Team CI, ed. UK: Health and Social Care Information Centre (HSIC), 2017.
6. Bardsley M, Blunt I, Davies S, et al. Is secondary preventive care improving? Observational study of 10-year trends in emergency admissions for conditions amenable to ambulatory care. *BMJ Open* 2013;3(1) doi: 10.1136/bmjopen-2012-002007 [published Online First: 2013/01/05]
7. Blunt I. Focus on preventable admissions. Trends in emergency admissions for ambulatory care sensitive conditions, 2001 to 2013. QualityWatch Focus, 2013.
8. Zhu CW, Cosentino S, Ornstein K, et al. Use and cost of hospitalization in dementia: Longitudinal results from a community-based study. *International Journal of Geriatric Psychiatry* 2015;30(8):833-41. doi: 10.1002/gps.4222
9. Bynum JPW, Rabins PV, Weller W, et al. The Relationship between a Dementia Diagnosis, Chronic Illness, Medicare Expenditures, and Hospital Use. *Journal of the American Geriatrics Society* 2004;52(2):187-94. doi: 10.1111/j.1532-5415.2004.52054.x
10. Phelan EA, Borson S, Grothaus L, et al. Association of incident dementia with hospitalizations. *JAMA - Journal of the American Medical Association* 2012;307(2):165-72. doi: 10.1001/jama.2011.1964
11. Lin PJ, Fillit HM, Cohen JT, et al. Potentially avoidable hospitalizations among Medicare beneficiaries with Alzheimer's disease and related disorders. *Alzheimer's and Dementia* 2013;9(1):30-38. doi: 10.1016/j.jalz.2012.11.002
12. Alzheimer's Association. Reducing potentially preventable hospitalisations for people living with Alzheimer's and other dementias 2017 [Available from: https://pdfs.semanticscholar.org/9448/37faa2cb818b66264a51d4a346aa8c1a0f93.pdf.
13. Han SD, Boyle PA, James BD, et al. Mild cognitive impairment is associated with poorer decision-making in community-based older persons. *J Am Geriatr Soc* 2015;63(4):676-83. doi: 10.1111/jgs.13346 [published Online First: 2015/04/09]
14. Ibrahim JE, Anderson LJ, MacPhail A, et al. Chronic disease self-management support for persons with dementia, in a clinical setting. *J Multidiscip Healthc* 2017;10:49-58. doi: 10.2147/JMDH.S121626 [published Online First: 2017/02/10]
15. Lin PJ, Rane PB, Fillit HM, et al. National estimates of potentially avoidable hospitalizations among medicare beneficiaries with Alzheimer's disease and related dementias. *Alzheimer's and Dementia* 2016;12(7):P253-P54.
16. Perera G, Broadbent M, Callard F, et al. Cohort profile of the South London and Maudsley NHS Foundation Trust Biomedical Research Centre (SLaM BRC) Case

For peer review only - http://bmjopen.bmj.com/site/about/guidelines.xhtml

Register: current status and recent enhancement of an Electronic Mental Health Record-derived data resource. *BMJ open* 2016;6(3):e008721.
17. Jackson RG, Patel R, Jayatilleke N, et al. Natural language processing to extract symptoms of severe mental illness from clinical text: the Clinical Record Interactive Search Comprehensive Data Extraction (CRIS-CODE) project. *BMJ open* 2017;7(1):e012012.
18. NHS Digital. Hospital Episode Statistics (HES) 2019 [Available from: https://digital.nhs.uk/data-and-information/data-tools-and-services/data-services/hospital-episode-statistics accessed 13/01/2019 2019.
19. Canuto A, Weber K, Gold G, et al. Structured assessment of mental health status in psychogeriatrics: validity of the French HoNOS65+. *Can J Psychiatry* 2007;52(1):37-45. doi: 10.1177/070674370705200107 [published Online First: 2007/04/21]
20. Ministry of Housing Communities and Local Government. The English Indices of Deprivation 2019. In: Ministry of Housing CaLG, ed., 2019.
21. NICE. Donepezil, galantamine, rivastigmine and memantine for the treatment of dementia. Health technology appraisal 2018 update, 2018.
22. Williams R. Understanding and interpreting generalized ordered logit models. *The Journal of Mathematical Sociology* 2016;40:7-20.
23. Maust DT, Kim HM, Chiang C, et al. Predicting Risk of Potentially Preventable Hospitalization in Older Adults with Dementia. *Journal of the American Geriatrics Society* 2019;67(10):2077-84. doi: 10.1111/jgs.16030
24. Natalwala A, Potluri R, Uppal H, et al. Reasons for hospital admissions in dementia patients in Birmingham, UK, during 2002-2007. *Dementia and Geriatric Cognitive Disorders* 2008;26(6):499-505. doi: 10.1159/000171044
25. Rao A, Suliman A, Vuik S, et al. Outcomes of dementia: Systematic review and meta-analysis of hospital administrative database studies. *Arch Gerontol Geriatr* 2016;66:198-204. doi: 10.1016/j.archger.2016.06.008
26. Hsu CC, Hsu YC, Chang KH, et al. Association of Dementia and Peptic Ulcer Disease: A Nationwide Population-Based Study. *Am J Alzheimers Dis Other Demen* 2016;31(5):389-94. doi: 10.1177/1533317515617546 [published Online First: 2016/01/24]
27. Janssens JP. Pneumonia in the elderly (geriatric) population. *Curr Opin Pulm Med* 2005;11(3):226-30. [published Online First: 2005/04/09]
28. Schnelle JF, Leung FW. Urinary and fecal incontinence in nursing homes. *Gastroenterology* 2004;126(1 Suppl 1):S41-7. [published Online First: 2004/02/24]
29. Palmer JL, Metheny NA. Preventing aspiration in older adults with dysphagia. *Am J Nurs* 2008;108(2):40-8. doi: 10.1097/01.NAJ.0000308961.99857.33 [published Online First: 2008/01/30]
30. Ninan S, Walton C, Barlow G. Investigation of suspected urinary tract infection in older people. *BMJ : British Medical Journal* 2014;349:g4070. doi: 10.1136/bmj.g4070
31. Burkett E, Carpenter CR, Arendts G, et al. Diagnosis of urinary tract infection in older persons in the emergency department: To pee or not to pee, that is the question. *Emerg Med Australas* 2019;31(5):856-62. doi: 10.1111/1742-6723.13376 [published Online First: 2019/09/04]
32. Mayne S, Bowden A, Sundvall PD, et al. The scientific evidence for a potential link between confusion and urinary tract infection in the elderly is still confusing - a

For peer review only - http://bmjopen.bmj.com/site/about/guidelines.xhtml

systematic literature review. *BMC Geriatr* 2019;19(1):32. doi: 10.1186/s12877-019-1049-7 [published Online First: 2019/02/06]
33. Knox S, Downer B, Haas A, et al. Dementia Severity Associated With Increased Risk of Potentially Preventable Readmissions During Home Health Care. *Journal of the American Medical Directors Association* 2019 doi: 10.1016/j.jamda.2019.09.012
34. Sachs GA, Carter R, Holtz LR, et al. Cognitive impairment: an independent predictor of excess mortality: a cohort study. *Ann Intern Med* 2011;155(5):300-8. doi: 10.7326/0003-4819-155-5-201109060-00007 [published Online First: 2011/09/07]
35. NHS. Pneumococcal Polysaccharide Vaccine (PPV) coverage report, England, April 2018 to March 2019. In: England PH, ed., 2019.
36. Marrie TJ. Community-acquired pneumonia in the elderly. *Clinical Infectious Diseases* 2000;31(4):1066-78. doi: 10.1086/318124
37. Chong CP, Street PR. Pneumonia in the elderly: a review of the epidemiology, pathogenesis, microbiology, and clinical features. *South Med J* 2008;101(11):1141-5; quiz 32, 79. doi: 10.1097/SMJ.0b013e318181d5b5 [published Online First: 2008/12/18]
38. Fillenbaum G, Heyman A, Peterson BL, et al. Use and cost of hospitalization of patients with AD by stage and living arrangement: CERAD XXI. *Neurology* 2001;56(2):201-6. [published Online First: 2001/02/13]
39. Voisin T, Andrieu S, Cantet C, et al. Predictive factors of hospitalizations in Alzheimer's disease: A two-year prospective study in 686 patients of the REAL.FR study. *Journal of Nutrition, Health and Aging* 2010;14(4):288-91. doi: 10.1007/s12603-010-0063-4
40. Afonso-Argilés FJ, Meyer G, Stephan A, et al. Emergency department and hospital admissions among people with dementia living at home or in nursing homes: results of the European RightTimePlaceCare project on their frequency, associated factors and costs. *BMC Geriatr* 2020;20(1):453. doi: 10.1186/s12877-020-01835-x [published Online First: 2020/11/07]
41. Packer R, Ben Shlomo Y, Whiting P. Can non-pharmacological interventions reduce hospital admissions in people with dementia? A systematic review. *PLOS ONE* 2019;14(10):e0223717. doi: 10.1371/journal.pone.0223717
42. Eyles E, Redaniel MT, Purdy S, et al. Associations of GP practice characteristics with the rate of ambulatory care sensitive conditions in people living with dementia in England: an ecological analysis of routine data. *BMC Health Serv Res* 2021;21(1):613. doi: 10.1186/s12913-021-06634-7 [published Online First: 2021/06/30]
43. Amjad H, Carmichael D, Austin AM, et al. Continuity of care and health care utilization in older adults with dementia in fee-for-service medicare. *JAMA Internal Medicine* 2016;176(9):1371-78. doi: 10.1001/jamainternmed.2016.3553
44. NHS England. Dementia diagnosis monthly workbook: Secondary dementia diagnosis monthly workbook 2017, 2017.
45. Shepherd H, Livingston G, Chan J, et al. Hospitalisation rates and predictors in people with dementia: A systematic review and meta-analysis. *BMC Medicine* 2019;17(1) doi: 10.1186/s12916-019-1369-7

For peer review only - http://bmjopen.bmj.com/site/about/guidelines.xhtml

Supplementary Table 1: Ambulatory care sensitive conditions*

ACSC	ICD code position		ICD-10 codes
	Main analysis	Sensitivity analysis	
**Angina**	Principal	1º or 2º	I20, I200, I201, I208, I209, I240, I248, I249, I25, I250, I251, I252, I253, I254, I255, I256, I258, I259
**Asthma**	Principal	1º or 2º	J45, J450, J451, J458, J459, J46, J46X
**Cellulitis**	Principal	1º or 2º	L03, L030, L031, L032, L033, L038, L039, L04, L040, L041, L042, L043, L048, L049, L08, L080, L081, L088, L089, L88, L88X, L980, L983
**Congestive heart failure**	Principal	1º or 2º	I110, I13, I130, I131, I132, I139, I50, I500, I501, I509, J81, J81X
**Convulsions and epilepsy**	Principal	1º or 2º	G40, G400, G401, G402, G403, G404, G405, G406, G407, G408, G409, G41, G410, G411, G412, G418, G419, O15, R56, R560, R568
**COPD**	Principal	1º or 2º	J20, J41, J410, J411, J418, J42, J42X, J43, J430, J431, J432, J438, J439, J44, J440, J441, J448, J449, J47, J47X
**Dehydration**	Principal	1º or 2º	E86, E86X
**Dental conditions**	Principal	1º or 2º	A690, K02, K020, K021, K022, K023, K024, K028, K029, K03, K030, K031, K032, K033, K034, K035, K036, K037, K038, K039, K04, K040, K041, K042, K043, K044, K045, K046, K047, K048, K049, K05, K050, K051, K052, K053, K054, K055, K056, K06, K060, K061, K062, K068, K069, K08, K080, K081, K082, K083, K088, K089, K09, K090, K091, K092, K098, K099, K12, K120, K121, K122, K13, K130, K131, K132, K133, K134, K135, K136, K137
**Diabetes complications**	Any	Any	E10, E100, E101, E102, E103, E104, E105, E106, E107, E108, E11, E110, E111, E112, E113, E114, E115, E116, E117, E118, E12, E120, E121, E122, E123, E124, E125, E126, E127, E128, E13, E130, E131, E132, E133, E134, E135, E136, E137, E138, E14, E140, E141, E142, E143, E144, E145, E146, E147, E148
**Ear, nose and throat infections**	Principal	1º or 2º	H66, H660, H661, H662, H663, H664, H669, H67, H670, H671, H678, J02, J020, J028, J029, J03, J030, J038, J039, J06, J060, J068, J069, J312
**Gangrene**	Any	Any	R02
**Gastroenteritis**	Principal	1º or 2º	K522, K528, K529
**Hypertension**	Principal	1º or 2º	I10, I10X, I119, I129, I139
**Influenza**	Any	Any	J10, J100, J101, J108, J11, J110, J111, J118
**Iron deficiency anaemia**	Principal	1º or 2º	D50, D501, D508, D509
**Nutritional deficiencies**	Principal	1º or 2º	E40, E40X, E41, E41X, E42, E42X, E43, E43X, E55, E550, E559, E64, E640, E641, E642, E643, E648, E649
**Nutritional deficiencies OR other nutritional deficiency anaemia**	Principal	1º or 2º	D51, D510, D511, D512, D513, D518, D519
**Other vaccine preventable**	Any	Any	A35, A35X, A36, A360, A361, A362, A363, A368, A369, A37, A370, A390, A80, A801, A802, A803, A804, A809, B05, B050, B051, B052, B053, B054, B058, B059, B06, B060, B068, B069, B16, B160, B161, B162, B169, B180, B181, B26, B260, B261, B262, B263, B268, B269, G000, G001, M010, M014
**Pelvic inflammatory disease**	Principal	1º or 2º	N70, N700, N701, N709, N73, N730, N731, N732, N733, N734, N735, N736, N738, N739, N74, N740, N741, N742, N743, N744, N748
**Perforated/bleeding ulcer**	Principal	1º or 2º	K25, K250, K251, K252, K254, K255, K256, K26, K260, K261, K262, K264, K265, K266, K27, K270, K271, K272, K274, K275, K276, K28, K280, K281, K282, K284, K286
**Pneumonia**	Any	Any	J13, J14, J153, J154, J157, J159, J168, J18, J180, J181, J182, J188, J189
**Tuberculosis**	Principal	1º or 2º	A15, A150, A151, A152, A153, A154, A155, A156, A157, A158, A159, A16, A160, A161, A162, A163, A164, A165, A167, A168, A169, A19, A190, A191, A192, A198, A199
**Urinary tract infection /Pyelonephritis**	Principal	1º or 2º	N10, N10X, N11, N110, N111, N118, N119, N12, N12X, N136, N30, N300, N301, N302, N303, N304, N308, N309, N390

ACSC: ambulatory care sensitive condition

*This table was created by the authors, however, the ACSCs, the respective ICD-10 codes and their position were initially based on those published by Bardsley *et al*, 2013. Codes were augmented with those published by the UK Health and Social Care Information Centre and by the authors. Deviations from the approach of Bardsley *et al* comprised of: i) cystitis codes were added to the UTI category; ii) O15 (eclampsia) was removed from the convulsions and epilepsy category. The approach used in the sensitivity was determined by the authors.

For peer review only

Supplementary Table 2: Results from stepwise-selected generalised ordinal logistic regression model for severity of dementia (sensitivity analysis *)

	Moderate/severe vs. mild dementia		Severe vs. mild/moderate dementia	
	**OR (95% CI)**	**p**	**OR (95% CI)**	**p**
**Sex (male reference)**	1.09 (0.97, 1.21)	0.141	1.09 (0.97, 1.21)	0.141
**Time since first presentation at SLaM**	1.13 (1.08, 1.18)	<0.001	1.13 (1.08, 1.18)	<0.001
**Age group at index date**	1.21 (1.11, 1.31)	<0.001	0.99 (0.89, 1.11)	0.856
**Ethnic group (European reference)**				
	1.21 (1.06, 1.38)	0.005	1.21 (1.06, 1.38)	0.005
**Dementia diagnostic group (AD reference)**				
Vascular	1.33 (1.15, 1.56)	<0.001	1.02 (0.85, 1.24)	0.816
Mixed	1.15 (0.95, 1.4)	0.163	0.8 (0.61, 1.05)	0.107
Other	1.11 (0.98, 1.27)	0.112	1.11 (0.98, 1.27)	0.112
**Deprivation score**	1.12 (1.06, 1.17)	<0.001	1.12 (1.06, 1.17)	<0.001
**Convulsions**	1.58 (0.96, 2.58)	0.07	1.58 (0.96, 2.58)	0.07
**Iron deficiency anaemia**	1.23 (0.63, 2.4)	0.542	0.25 (0.06, 1.05)	0.058
**Perforated/bleeding ulcer**	6.92 (1.56, 30.77)	0.011	6.92 (1.56, 30.77)	0.011
**Pneumonia**	1.38 (1.12, 1.69)	0.002	1.38 (1.12, 1.69)	0.002
**UTI pyelonephritis**	1.29 (1.1, 1.51)	0.002	1.29 (1.1, 1.51)	0.002

Goodness of fit: LR Chi square 143.3, $p<0.0001$
ACSC: ambulatory care sensitive condition
*Criteria were relaxed to include diagnoses in either of the first two positions for ACSCs that were initially defined based on a diagnosis in the primary position only.

For peer review only

STROBE 2007 (v4) checklist of items to be included in reports of observational studies in epidemiology*
Checklist for cohort, case-control, and cross-sectional studies (combined)

Section/Topic	Item #	Recommendation	Reported on page #
**Title and abstract**	1	(*a*) Indicate the study's design with a commonly used term in the title or the abstract	1
		(*b*) Provide in the abstract an informative and balanced summary of what was done and what was found	2
**Introduction**			
Background/rationale	2	Explain the scientific background and rationale for the investigation being reported	5
Objectives	3	State specific objectives, including any pre-specified hypotheses	5
**Methods**			
Study design	4	Present key elements of study design early in the paper	6
Setting	5	Describe the setting, locations, and relevant dates, including periods of recruitment, exposure, follow-up, and data collection	6
Participants	6	(*a*) *Cohort study*—Give the eligibility criteria, and the sources and methods of selection of participants. Describe methods of follow-up<br>*Case-control study*—Give the eligibility criteria, and the sources and methods of case ascertainment and control selection. Give the rationale for the choice of cases and controls<br>*Cross-sectional study*—Give the eligibility criteria, and the sources and methods of selection of participants	6
		(*b*) *Cohort study*—For matched studies, give matching criteria and number of exposed and unexposed<br>*Case-control study*—For matched studies, give matching criteria and the number of controls per case	
Variables	7	Clearly define all outcomes, exposures, predictors, potential confounders, and effect modifiers. Give diagnostic criteria, if applicable	8-9
Data sources/ measurement	8*	For each variable of interest, give sources of data and details of methods of assessment (measurement). Describe comparability of assessment methods if there is more than one group	*8-9*
Bias	9	Describe any efforts to address potential sources of bias	
Study size	10	Explain how the study size was arrived at	N/A
Quantitative variables	11	Explain how quantitative variables were handled in the analyses. If applicable, describe which groupings were chosen and why	8-9
Statistical methods	12	(*a*) Describe all statistical methods, including those used to control for confounding	10-11
		(*b*) Describe any methods used to examine subgroups and interactions	10-11
		(*c*) Explain how missing data were addressed	N/A
		(*d*) *Cohort study*—If applicable, explain how loss to follow-up was addressed<br>*Case-control study*—If applicable, explain how matching of cases and controls was addressed	

For peer review only

		Cross-sectional study—If applicable, describe analytical methods taking account of sampling strategy	
		(*e*) Describe any sensitivity analyses	9
**Results**			
Participants	13*	(a) Report numbers of individuals at each stage of study—eg numbers potentially eligible, examined for eligibility, confirmed eligible, included in the study, completing follow-up, and analysed	
		(b) Give reasons for non-participation at each stage	
		(c) Consider use of a flow diagram	
Descriptive data	14*	(a) Give characteristics of study participants (eg demographic, clinical, social) and information on exposures and potential confounders	12
		(b) Indicate number of participants with missing data for each variable of interest	N/A
		(c) *Cohort study*—Summarise follow-up time (eg, average and total amount)	
Outcome data	15*	*Cohort study*—Report numbers of outcome events or summary measures over time	15
		Case-control study—Report numbers in each exposure category, or summary measures of exposure	
		Cross-sectional study—Report numbers of outcome events or summary measures	
Main results	16	(*a*) Give unadjusted estimates and, if applicable, confounder-adjusted estimates and their precision (eg, 95% confidence interval). Make clear which confounders were adjusted for and why they were included	16
		(*b*) Report category boundaries when continuous variables were categorized	
		(*c*) If relevant, consider translating estimates of relative risk into absolute risk for a meaningful time period	
Other analyses	17	Report other analyses done—eg analyses of subgroups and interactions, and sensitivity analyses	
**Discussion**			
Key results	18	Summarise key results with reference to study objectives	19
Limitations	19	Discuss limitations of the study, taking into account sources of potential bias or imprecision. Discuss both direction and magnitude of any potential bias	21
Interpretation	20	Give a cautious overall interpretation of results considering objectives, limitations, multiplicity of analyses, results from similar studies, and other relevant evidence	22
Generalisability	21	Discuss the generalisability (external validity) of the study results	21
**Other information**			
Funding	22	Give the source of funding and the role of the funders for the present study and, if applicable, for the original study on which the present article is based	23

*Give information separately for cases and controls in case-control studies and, if applicable, for exposed and unexposed groups in cohort and cross-sectional studies.
Note: An Explanation and Elaboration article discusses each checklist item and gives methodological background and published examples of transparent reporting. The STROBE checklist is best used in conjunction with this article (freely available on the Web sites of PLoS Medicine at http://www.plosmedicine.org/, Annals of Internal Medicine at http://www.annals.org/, and Epidemiology at http://www.epidem.com/). Information on the STROBE Initiative is available at www.strobe-statement.org.

For peer review only

BMJ Open

POTENTIALLY AVOIDABLE CAUSES OF HOSPITALISATION IN PEOPLE WITH DEMENTIA: CONTEMPORANEOUS ASSOCIATIONS BY STAGE OF DEMENTIA IN A SOUTH LONDON CLINICAL COHORT

Journal:	*BMJ Open*
Manuscript ID	bmjopen-2021-055447.R2
Article Type:	Original research
Date Submitted by the Author:	04-Mar-2022
Complete List of Authors:	Gungabissoon, Usha; GSK, Epidemiology (Value Evidence and Outcomes); King's College London Institute of Psychiatry Psychology and Neuroscience, Psychological Medicine<br>Perera, Gayan; King's College London Institute of Psychiatry Psychology and Neuroscience, Psychological Medicine<br>Galwey, Nicholas; GSK, Research Statistics<br>Stewart, Robert; King's College London Institute of Psychiatry Psychology and Neuroscience, Psychological Medicine; South London and Maudsley NHS Foundation Trust
**Primary Subject Heading**:	Epidemiology
Secondary Subject Heading:	Geriatric medicine, Health services research, Mental health
Keywords:	Dementia < NEUROLOGY, EPIDEMIOLOGY, Old age psychiatry < PSYCHIATRY

SCHOLARONE™
Manuscripts

I, the Submitting Author has the right to grant and does grant on behalf of all authors of the Work (as defined in the below author licence), an exclusive licence and/or a non-exclusive licence for contributions from authors who are: i) UK Crown employees; ii) where BMJ has agreed a CC-BY licence shall apply, and/or iii) in accordance with the terms applicable for US Federal Government officers or employees acting as part of their official duties; on a worldwide, perpetual, irrevocable, royalty-free basis to BMJ Publishing Group Ltd ("BMJ") its licensees and where the relevant Journal is co-owned by BMJ to the co-owners of the Journal, to publish the Work in this journal and any other BMJ products and to exploit all rights, as set out in our licence.

The Submitting Author accepts and understands that any supply made under these terms is made by BMJ to the Submitting Author unless you are acting as an employee on behalf of your employer or a postgraduate student of an affiliated institution which is paying any applicable article publishing charge ("APC") for Open Access articles. Where the Submitting Author wishes to make the Work available on an Open Access basis (and intends to pay the relevant APC), the terms of reuse of such Open Access shall be governed by a Creative Commons licence – details of these licences and which Creative Commons licence will apply to this Work are set out in our licence referred to above.

Other than as permitted in any relevant BMJ Author's Self Archiving Policies, I confirm this Work has not been accepted for publication elsewhere, is not being considered for publication elsewhere and does not duplicate material already published. I confirm all authors consent to publication of this Work and authorise the granting of this licence.

For peer review only

WORD COUNT: 4224

TITLE: POTENTIALLY AVOIDABLE CAUSES OF HOSPITALISATION IN PEOPLE WITH DEMENTIA: CONTEMPORANEOUS ASSOCIATIONS BY STAGE OF DEMENTIA IN A SOUTH LONDON CLINICAL COHORT

Authors:

Usha Gungabissoon[1,2], M.Sc.; Gayan Perera[1], Ph.D.; Nicholas Galwey[3], Ph.D.; Robert Stewart[1,4], M.D., FRCPsych

[1]Institute of Psychiatry, Psychology and Neuroscience (IOPPN), King's College London, London, United Kingdom (UK)

[2]Epidemiology, Value, Evidence and Outcomes, Global Medical, GlaxoSmithKline (GSK) R&D, London, UK

[3]Research Statistics, GSK R&D, London, UK

[4]South London and Maudsley NHS Foundation Trust, London, UK

For peer review only

For peer review only - http://bmjopen.bmj.com/site/about/guidelines.xhtml

Corresponding author: Usha Gungabissoon

Department of Psychological Medicine (PO84), Institute of Psychiatry, Psychology and Neuroscience, King's College London. De Crespigny Park, London SE5 8AF

Email: usha.2.gungabissoon@gsk.com

Phone: +442080476878

For peer review only

For peer review only - http://bmjopen.bmj.com/site/about/guidelines.xhtml

ABSTRACT (word count 300)

Objectives

To estimate the frequency of all-cause and ambulatory care sensitive condition (ACSCs)-related hospitalisations amongst individuals with dementia. In addition, to investigate differences by stage of dementia based on recorded cognitive function.

Setting

Data from a large London dementia care clinical case register, linked to a national hospitalisation database.

Participants

Individuals aged ≥65 years with a confirmed dementia diagnosis with recorded cognitive function.

Outcome measures

Acute general hospital admissions were evaluated within 6 months of a randomly selected cognitive function score in patients with a clinical diagnosis of dementia. To evaluate associations between ACSC-related hospital admissions (overall and individual ACSCs) and stage of dementia, an ordinal regression was performed, modelling stage of dementia as the dependant variable (to facilitate efficient model selection, with no implication concerning the direction of causality).

Results

Of 5,294 people with dementia, 2,993 (56.5%) had at least one hospitalisation during a 12-month period of evaluation, and 1,192 (22.5%) had an ACSC-related admission. Proportions with an all-cause or ACSC-related hospitalisation were greater in the groups with more advanced dementia (all-cause 53.9%, 57.1% and 60.9%, p 0.002; ACSC-related 19.5%, 24.0% 25.3%, p <0.0001in the mild, moderate and severe groups respectively). An ACSC-related admission was associated with 1.3-fold (95% CI 1.1-1.5) increased odds of more severe dementia after adjusting for demographic factors. Concerning admissions for individual ACSCs, the most common ACSC was UTI/pyelonephritis (9.8% of hospitalised patients) followed by pneumonia (7.1%); in an adjusted model, these were each associated with 1.4-fold increased odds of more severe dementia (95% CI, 1.2-1.7 and 1.1-1.7 respectively).

For peer review only - http://bmjopen.bmj.com/site/about/guidelines.xhtml

For peer review only

Conclusions

Potentially avoidable hospitalisations were common in people with dementia, particularly in those with greater cognitive impairment. Our results call for greater attention to the extent of cognitive status impairment, and not just dementia diagnosis, when evaluating measures to reduce the risk of potentially avoidable hospitalisations.

Key words: dementia, epidemiology, healthcare utilisation, avoidable hospitalisation, ambulatory care sensitive

For peer review only

For peer review only - http://bmjopen.bmj.com/site/about/guidelines.xhtml

Strengths and limitations of this study

- Through linkage of a catchment specialist healthcare case register to a national hospitalisation database, our study benefited from near-complete outcome ascertainment enabling us to evaluate potentially avoidable causes of hospitalisation in a large sample of clinically confirmed cases of dementia.
- Severity of dementia was ascertained from routinely administered cognitive assessments, making use of natural language processing algorithms with established validity.
- Reasons for hospitalisation were derived from the discharge diagnoses recorded for last episode of the hospital admission, meaning it was not possible to distinguish complications arising during the hospital stay from reasons for the initial admission.
- Data were obtained from single service provider in an urban catchment area, which may limit the generalisability of our findings to other settings.
- The analyses did not account for comorbidities, use of medications or living arrangement (such as home versus care home); furthermore, dementia stage and hospitalisations were measured contemporaneously, meaning that conclusions about causation should not be drawn.

For peer review only

INTRODUCTION

Dementia represents a significant challenge for health services. For example, in the UK National Health Service (NHS) an estimated 25% of acute beds are occupied by people with dementia[1] and recent analyses have shown a 35% increase in the number of emergency hospital admissions for people with dementia in England over the past five years.[2] In 2016, the global economic costs caused by dementia approached $1 trillion, with the highest economic burden seen in Europe and North America.[3] Major contributing factors to costs include disease severity and higher rates of hospitalisation in people with dementia relative to older people without dementia.[4]

Whilst some hospitalisations are necessary and expected, others are potentially avoidable. Ambulatory care sensitive conditions (ACSCs) are conceptualised as chronic and acute health conditions such as heart failure, pneumonia, pyelonephritis and complications of diabetes, for which effective primary and community care may avoid the need for hospital admission by preventing the onset of these diseases, controlling the acute episodic illness, or managing the chronic condition more effectively.[5 6] ACSC-related admissions, for example, are used to assess how the NHS is performing in England.[7]

For peer review only - http://bmjopen.bmj.com/site/about/guidelines.xhtml

Preventing hospitalisation is of particular importance in people with dementia; hospitalisation is associated with poorer outcomes including prolonged hospital stays and increased risk of complications relative to people without dementia.[8-11] Further, hospitalisation itself can trigger distress, or confusion which can contribute to a decline in functioning creating new care challenges, or can intensify the existing burden of care.[12]

Even at early stages of cognitive impairment, a person's ability to make health care decisions can be altered, [13] including changes in health-seeking behaviours and management of existing conditions [14]. This could explain why the age-adjusted risk of potentially avoidable hospitalisations is greater in people with dementia than those without. [9 10] There is also some evidence suggesting that the risk of potentially avoidable hospitalisations is greater in people with later-stage dementia.[8 15] However, previous studies have been small, used non-representative convenience samples or relatively crude methods to evaluate cognitive deficits. Understanding which potentially avoidable causes of hospitalisation predominate at later stages of dementia can help to tailor healthcare needs in this population in order to prevent the need for hospitalisation.

For peer review only - http://bmjopen.bmj.com/site/about/guidelines.xhtml

In the present study, we used data drawn from a mental healthcare case register linked to national hospitalisation episodes, to describe ACSC-related hospitalisations, a proxy for potentially avoidable hospitalisations, amongst a large sample of patients with dementia. Specifically, we sought first to investigate whether ACSC-related hospital admission was associated with more advanced stages of dementia, and second to explore whether hospitalisations for specific ACSCs were associated with more advanced dementia stage.

For peer review only

For peer review only - http://bmjopen.bmj.com/site/about/guidelines.xhtml

METHODS

Study design

This study investigated acute general hospital admissions around the time of a given cognitive function score (index date) in a sample of patients with a clinical diagnosis of dementia using linked clinical datasets.

Data sources and sample

The study sample comprised of residents within the South London and Maudsley NHS Foundation Trust (SLaM) who had received a dementia diagnosis in specialist services. The trust is a provider of mental healthcare, dementia assessment and management, for a south London catchment of >1.2 million residents in Lambeth, Lewisham, Croydon and Southwark; EHRs have been implemented across all SLaM services since 2006.[16] The Clinical Record Interactive Search (CRIS) data resource, used to identify dementia cases, provides research access to anonymised electronic health records (EHRs) ; it allows both structured and unstructured data to be abstracted from patient records based on interactions within secondary care mental health services within SLaM.

For peer review only - http://bmjopen.bmj.com/site/about/guidelines.xhtml

Routine diagnoses are recorded using the International Classification of Diseases 10th edition (ICD-10) coding system, and are supplemented in CRIS by a natural language processing (NLP) to abstract measures of cognitive impairment.[16 17] Hospital outcomes (hospital admissions and their causes) for patients with dementia were obtained from the a linkage with the Hospital Episode Statistics (HES) database. HES contains details of all inpatient admissions at NHS hospitals in England.[18] Discharge diagnoses are also recorded as ICD-10 codes and are available for each hospitalisation episode. HES data up to 31st March 2013 were available at the time of analysis.

For peer review only

Study population

Patients with a diagnosis of dementia first recorded in SLaM between 1 January 2006 and 30th September 2012, and aged ≥65 years at diagnosis, were identified on CRIS. Dementia was defined on the presence of F00*, F01*, F02*or F03* ICD diagnosis codes recorded up to the sixth position in the structured data. Eligible patients were those who had at least one cognitive

For peer review only - http://bmjopen.bmj.com/site/about/guidelines.xhtml

assessment (from structured data or free text using NLP) recorded: either a Mini Mental State Examination score (MMSE) or a cognitive impairment score from the Health of the Nation Outcome Scales (HoNOS). HoNOS is a clinician-rated instrument usually completed on a structured field within the EHR at first assessment, which contains subscales rated 0 (no problem) to 4 (severe/very severe problem); its cognitive scale correlates with MMSE measurement.[19] To exclude patients with potential mild cognitive impairment rather than dementia, the sample was further restricted to those with an MMSE score of <28 or HoNOS cognitive impairment score >1. In addition, we excluded patients who received their first dementia diagnosis from an acute hospital liaison service; these reflected cases where the recording of a dementia diagnosis might have been precipitated by a hospitalisation and by definition be a group with significant hospitalised physical disorders, therefore potentially biasing any associations between dementia and comorbidity.

For each eligible patient, we randomly selected a single measure of cognition recorded in the patient's clinical record using a random number generator in Microsoft Excel; the date for the measure was assigned as the index date. This process was applied hierarchically: if several MMSE scores were recorded over time for a given patient, then one was randomly selected;

For peer review only - http://bmjopen.bmj.com/site/about/guidelines.xhtml

for those individuals without an MMSE measurement, a random Health of the Nation Outcome Scales (HoNOS) cognitive impairment subscale was selected in the same manner. The randomly selected score for each patient was used to categorise the stage of dementia into mild, moderate or severe on the date of the cognitive assessment as described below.

Measurements

The following demographic information was obtained from CRIS: age at diagnosis of dementia, sex, ethnicity (White-European, other, not specified), dementia diagnostic group (Alzheimer's, vascular, mixed or other) and Index of Multiple Deprivation (IMD) score, a widely used measure of relative neighbourhood deprivation.[20] IMD is derived from Census data and calculated for clusters of adjacent unit postcodes in England known as Lower layer Super Output Areas (LSOA), standard geographic units with around 1500 residents; a higher IMD score indicates a more deprived area.

To classify dementia stage at the index date, we used cut-offs similar to those used by others[21], defining MMSE scores of >21-27, 10-20, and <10, or a HoNOS cognitive impairment subscale score of 2, 3, and 4, as representing mild, moderate, or severe dementia, respectively.

For peer review only - http://bmjopen.bmj.com/site/about/guidelines.xhtml

Acute general hospital inpatient admissions were obtained for the 6-month period either side of the index date, i.e., over a 12-month period. Hospitalisations were defined from HES episodes, combining contiguous episodes (i.e., where start and end dates were on the same day); in the context of this analysis, an admission, or spell, is defined as a continuous period spent as a patient in hospital. Four-digit ICD-10 discharge diagnoses recorded during the last episode of each hospital admission were obtained (up to twenty diagnoses may be recorded as part of a hospital episode).

Ambulatory care sensitive conditions (ACSC) were identified based on the presence of specific ICD-10 codes and their position in the record, using a modification of the approach used by Bardsley *et al* and the UK Health and Social Care Information Centre; for example, angina is based on the presence of specific ICD-10 codes in the primary position, whereas pneumonia is defined based on the presence of a relevant code in any position.[5 6] Deviations from the published approach comprised the following: i) for the UTI category, we deemed cystitis to be relevant and therefore included relevant codes in this group; ii) we removed O15 (eclampsia) from the convulsions and epilepsy category as used by Bardsley. Each ACSC was specified

For peer review only - http://bmjopen.bmj.com/site/about/guidelines.xhtml

individually as a binary variable to flag the presence of a hospitalisation with that specific condition. Given that diagnoses may not be recorded in the order of clinical importance in the real-world setting, as a sensitivity analysis, we relaxed the requirement for conditions which were based on a diagnosis in the primary position only, to include diagnoses in either of the first two positions. **Supplementary table 1** lists the ACSCs, the ICD codes and the position of the ICD code.

Additional covariates included binary variables to indicate an all-cause or any ACSC-related hospitalisation during the 12-month observation period. Time since first recorded dementia diagnosis (a proxy for disease duration) in SLaM at index date was also obtained.

Statistical analyses

Microsoft Excel was used for data management, and Stata 15·0 was used for statistical analyses. Patient characteristics were described by stage of dementia; normally distributed variables were presented as mean ± standard deviation (SD) and non-normally distributed variables as median and upper and lower quartiles. For the bivariate analysis, comparison of continuous variables between dementia stages (mild, moderate, severe) was evaluated using

For peer review only

ANOVA, with the exception of number of inpatient admissions and time since presentation at SLaM which were evaluated by the Kruskal-Wallis test due to their non-normal distribution. For the relationship between each categorical variable and stage of dementia, a chi-squared test of association was performed, and percentage distributions presented.

To evaluate the association between any ACSC-related hospitalisation and contemporaneously measured stage of dementia, unadjusted, age-sex-adjusted and fully adjusted odds ratios (OR) were obtained from generalised ordinal logistic regression models,[22] with dementia stage (mild, moderate, severe) as the dependant variable. Including dementia stage as the outcome variable was deemed to be the most efficient approach to evaluate ACSC-related hospitalisations and specific causes of ACSC-related admissions as it allowed us to use the efficient, flexible approach of stepwise model selection, to evaluate the importance of the relationship with other variables. Because our aim was to assess co-temporal associations between stage and hospitalisations rather than causality, we considered that whether dementia stage was modelled as a response, or as an explanatory variable, was not critical.

To investigate whether specific causes of ACSC-related admissions were associated with dementia stage, a backward stepwise model selection was used to build a multivariable

For peer review only - http://bmjopen.bmj.com/site/about/guidelines.xhtml

generalised ordinal logistic regression model, with dementia stage as the dependant variable. We used the gologit2 STATA command with the autofit option to selectively relax the model, removing the proportional odds assumption for any variable for which this assumption was violated.[22] Sex, time from first dementia record at index date (years) and age-group at index date were deemed clinically relevant and were therefore forced into the model (i.e., retained regardless of their p-value). Variables with p<0.1 in the bivariate analysis were initially included in the stepwise model selection. During stepwise selection, variables with p <0.2 were retained in the model, and following exclusion of any variable, the variables already excluded were considered for re-inclusion. Collinearity was evaluated based on the Variance Inflation Factor (VIF) and tolerance for each variable; covariates with a VIF greater than 2.5 were removed one at a time, starting with variables with the largest VIF, and the impact on remaining covariates was assessed until no remaining variable had a VIF >2.5. Further, interactions between age-group and sex, and between age-group and dementia diagnostic group, were assessed for possible inclusion, based on the Akaike Information Criterion (AIC).

Patient and Public Involvement

Patients or the public were not involved in the design, or conduct, or reporting, or dissemination plans of our research.

For peer review only - http://bmjopen.bmj.com/site/about/guidelines.xhtml

For peer review only

For peer review only - http://bmjopen.bmj.com/site/about/guidelines.xhtml

RESULTS

In total, 5,294 patients with dementia met the study criteria and were included in the analysis sample. Almost two-thirds of the sample were female (N=3,393, 64.1%) and the mean age at the date of randomly chosen cognitive assessment was 82.5 years (SD 6.8). The proportions of patients with mild, moderate and severe dementia at these index dates were 37.3%, 46.4% and 16.1% respectively. Descriptive characteristics by stage of dementia are presented in **Table 1.**

Table 1: Descriptive analysis by dementia severity (N=5,294)

		Mild (N=1,979)	Moderate (N=2,461)	Severe (N=854)	All (N=5,294)	Test statistic [d.f] p value
**Age at index date N (%)**	65-74y	326 (16.5)	292 (11.9)	116 (13.6)	734 (13.9)	χ^2 [4] = 23.35 p <0.0001
	75-84y	892 (45.1)	1107 (45.0)	397 (46.5)	2396 (45.3)	
	85+	761 (38.5)	1062 (43.2)	341 (39.9)	2164 (40.9)	
**Mean age at index date (SD)**		81.9 (6.9)	82.9 (6.8)	82.4 (6.9)	82.5 (6.8)	F [2, 5291] = 11.96 p <0.0001
**Sex**	Female	1233 (62.3)	1615 (65.6)	545 (63.8)	3393 (64.1)	χ^2 [2]=5.29 p 0.071
	Male	746 (37.7)	846 (34.4)	309 (36.2)	1901 (35.9)	
**Ethnic group**	White-European	1614 (81.6)	1915 (77.8)	655 (76.7)	4184 (79.0)	χ^2 [4] = 9.54 p 0.008
	Other	337 (17.0)	502 (20.4)	172 (20.1)	1011 (19.1)	
	Not Specified	28 (1.4)	44 (1.8)	27 (3.2)	99 (1.9)	

For peer review only - http://bmjopen.bmj.com/site/about/guidelines.xhtml

**Deprivation score - mean (SD)**		26.02 (11.4)	27.46 (11.0)	28.1 (11.0)	27.02 (11.2)	F [2, 5260] = 13.75 p <0.0001
**Time (years) since first presentation at SLaM – median (IQR)**		0.31 (0.03 ,0.91)	0.31 (0.02 ,1.21)	0.31 (0.02 ,1.28)	0.31 (0.02 ,1.06)	H (2) = 1.04 p 0.596
**Dementia diagnostic group**	Alzheimer's	852 (43.1)	931 (37.8)	336 (39.34)	2119 (40.03)	χ^2 [6] = 25.81 p <0.0001
	Mixed	206 (10.4)	278 (11.3)	74 (8.7)	558 (10.5)	
	Vascular	385 (19.5)	602 (24.5)	197 (23.1)	1184 (22.4)	
	Other	536 (27.1)	650 (26.4)	247 (28.9)	1433 (27.1)	.

Percentages presented in parentheses unless otherwise specified

Test statistics: χ^2 Chi square test of association, F: One-way ANOVA, H: Kruskal-Wallis test.

d.f: degrees of freedom

All-cause and ACSC-related hospitalisations

Overall, 56.5% (N=2,993 of 5,294) of individuals analysed had at least one inpatient admission (of any cause) in the 6-month period either side of the index date, and this percentage was greater with more advanced dementia: 53.9% of patients with mild, 57.1% with moderate and 60.9% with severe dementia (χ^2 (d.f. 2) =12.47, p = 0.002) (**Table 2**).

Within the cohort, 22.5% had at least one ACSC-related hospitalisation (N=1,192/5,294) which accounted for almost 40% of patients with any hospital admission (N=1,192/2,993). ACSC-related hospitalisations were observed in 36.2%, 42.0% and 41.5% of hospitalised patients with mild, moderate and severe dementia respectively (χ^2 (d.f. 2) =9.25 p = 0.01). The most

For peer review only - http://bmjopen.bmj.com/site/about/guidelines.xhtml

common ACSC was UTI/pyelonephritis (9.8% of hospitalised patients) followed by pneumonia (7.1%), both of which tended to be more frequent with more severe dementia (p <0.0001 for both conditions). The mean number of ACSC admissions amongst hospitalised patients was highest in those with moderate (0.67, SD 1.04) compared to mild or severe dementia (0.57, SD 1.01 and 0.58, SD 0.85, F (2, 2990) = 3.33, p = 0.036).

For peer review only

For peer review only - http://bmjopen.bmj.com/site/about/guidelines.xhtml

Table 2: Hospital admissions by severity of dementia (N=5,294)

	Mild (N=1979)	Moderate (N=2461)	Severe (N=854)	All (N=5294)	Test statistic [d.f] p value
Inpatient admission +/- 6 months of index date, N (%)	1067 (53.9)	1406 (57.1)	520 (60.9)	2993 (56.5)	χ^2 [2] = 12.47 p 0.002
Number of all cause admissions per patient – Median (lower and upper quartile)*	2 (1,3)	2 (1,3)	2 (1,3)	2 (1,3)	H [2] = 3.42 p 0.181
Ambulatory care sensitive conditions N (% of all patients)					
Angina	27 (1.4)	28 (1.1)	5 (0.6)	60 (1.1)	χ^2 [2] = 3.2 p 0.199
Asthma	2 (0.1)	2 (0.1)	1 (0.1)	5 (0.1)	χ^2[2] = 0.10 p 0.951
Cellulitis	19 (1.0)	27 (1.1)	9 (1.1)	55 (1.0)	χ^2 [2] = 0.20 p 0.904
Congestive heart failure	28 (1.4)	38 (1.5)	10 (1.2)	76 (1.4)	χ^2 [2] = 0.63 p 0.729
Convulsion/epilepsy	16 (0.8)	23 (0.9)	12 (1.4)	51 (1.0)	χ^2 [2] = 2.26 p 0.322
COPD	28 (1.4)	40 (1.6)	8 (0.9)	76 (1.4)	χ^2 [2] = 2.13 p 0.344
Dehydration	8 (0.4)	20 (0.8)	7 (0.8)	35 (0.7)	χ^2[2] = 3.18 p 0.204
Dental	9 (0.5)	10 (0.4)	1 (0.1)	20 (0.4)	χ^2 [2] = 1.91 p 0.385
Diabetes complications	30 (1.5)	48 (2.0)	11 (1.3)	89 (1.7)	χ^2 [2] = 2.205 p 0.332
ENT infections	5 (0.3)	2 (0.1)	1 (0.1)	8 (0.2)	χ^2 [2] = 2.21 p 0.331
Gastroenteritis	21 (1.1)	20 (0.8)	3 (0.4)	44 (0.8)	χ^2 [2] = 3.67 p 0.16
Hypertension	3 (0.2)	3 (0.1)	-	6 (0.1)	χ^2 [2] = 1.24 p 0.538
Iron deficiency anaemia	6 (0.3)	15 (0.6)	2 (0.2)	23 (0.4)	χ^2 [2] = 3.32 p 0.19
Other vaccine preventable	2 (0.1)	-	-	2 (0.04)	χ^2 [2] = 3.35 p 0.187

For peer review only - http://bmjopen.bmj.com/site/about/guidelines.xhtml

**Perforated/bleeding ulcer**	-	3 (0.1)	3 (0.4)	6 (0.1)	χ^2 [2] = 6.53 p 0.038
**Pneumonia**	103 (5.2)	201 (8.2)	70 (8.2)	374 (7.1)	χ^2 [2] = 16.653 p <0.0001
**UTI pyelonephritis**	155 (7.8)	255 (10.4)	111 (13.0)	521 (9.8)	χ^2 [2] = 19.34 p <0.0001
**Any ACSC admission N (%)**					
**overall**	386 (19.5)	590 (24.0)	216 (25.2)	1192 (22.5)	χ^2 [2] = 17.06 p <0.0001
**amongst those with an inpatient admission**	386 (36.2)	590 (42.0)	216 (41.5)	1192 (39.8)	χ^2 [2] = 9.25 p 0.010
**Mean number of ACSC admissions per patient (SD)**					
**amongst those with an inpatient admission**	0.6 (1.0)	0.7 (1.0)	0.6 (0.9)	0.6 (1.0)	F [2, 2990] = 3.33 p 0.036

□ No admissions meeting the criteria for flu or gangrene identified

* amongst hospitalised

Test statistics: χ^2 Chi square test, F: One-way ANOVA, H: Kruskal-Wallis test

d.f: degrees of freedom

For peer review only

Associations between any ACSC-related hospitalisation and dementia stage

Any ACSC-related hospitalisation was significantly associated with more advanced dementia, i.e., an OR significantly greater than 1 for moderate/severe vs. mild, or severe vs. mild/moderate, in the unadjusted model (unadjusted OR 1.28, 95% CI 1.14, 1.45). The association remained significant in the fully adjusted model which accounted for age, sex, time since first presentation on SLaM, ethnic-group and neighbourhood deprivation (OR 1.27, 95% CI 1.12, 1.44 for both moderate/severe vs. mild and severe vs. mild/moderate) (**Table 3**).

Table 3: Unadjusted and adjusted ORs and 95% CIs for any ACSC-related admission by stage of dementia

	Severe/moderate vs. mild or Severe vs. mild/moderate□<br><br>OR (95% CI)
**Unadjusted**	1.28 (1.14, 1.45)
**Age, sex adjusted**	1.27 (1.13, 1.44)
**Fully adjusted***	1.27 (1.12, 1.44)

*Age-group, sex, time since first presentation on SLaM, ethnicity and IMD

□From a generalised ordinal logistic regression model: the same odds ratio applies to both comparisons as the proportional odds assumption is not violated.

Exploratory analysis: associations between specific ACSC-related hospitalisations and dementia stage

For peer review only - http://bmjopen.bmj.com/site/about/guidelines.xhtml

Variables included in the multivariable model after stepwise selection are summarised in **Table 4**. The goodness of fit of the final model, as measured by the likelihood ratio chi-square test, demonstrated that the model as a whole fitted significantly better than the null model (i.e., a model with no predictors; LR Chi square 144.6, p <0.0001). The output from the stepwise-selected regression model is presented in **Table 4**. The proportional odds assumption was violated for age-group, and dementia diagnostic group, meaning that the effect of these explanatory variables was not the same at the two dementia-severity boundaries.

The model indicated that, conditional on other explanatory variables, a greater time since first presentation at SLaM, higher IMD score (i.e., residence in a more deprived area) and "other" ethnicity were associated with greater odds of more severe dementia. Greater age was associated with greater odds of moderate/severe dementia relative to mild, but not of severe dementia relative to mild/moderate.

Considering specific conditions, perforated/bleeding ulcer, UTI/pyelonephritis and pneumonia hospitalisations were all significantly associated with greater odds of more advanced dementia, i.e., moderate/severe relative to mild or severe relative to mild/moderate. Although a strong

For peer review only - http://bmjopen.bmj.com/site/about/guidelines.xhtml

association between perforated/bleeding ulcer and dementia stage (OR 7.01 *p* 0.01) was observed, it is important to note that in terms of absolute frequency this condition was uncommon, being recorded in only 6 individuals. UTI/pyelonephritis and pneumonia were also associated with higher odds of more severe dementia (OR 1.38 *p* <0.0001, OR 1.38 *p* 0.002 respectively); this was consistent across dementia severity categories, i.e., the ORs and p-values apply to both cut-points. Gastroenteritis-related admissions had an inverse relationship with dementia-stage, namely that admission was associated with a lower odds of advanced dementia stages: however, this was of borderline significance (OR 0.57 *p* 0.06).

Table 4: Results from stepwise-selected partial generalised ordinal logistic regression model for severity of dementia (N=5,294)*

Explanatory variable	Moderate/severe vs. mild dementia		Severe vs. mild/moderate Dementia	
	OR (95% CI)	p	OR (95% CI)	p
Sex (male reference)	1.07 (0.96, 1.2)	0.203	1.07 (0.96, 1.2)	0.203
Time since first presentation at SLaM	1.13 (1.08, 1.19)	<0.0001	1.13 (1.08, 1.19)	<0.0001
Age group at index date (65-74 years reference)				
	1.36 (1.15, 1.62)	<0.0001	1.04 (0.82, 1.31)	0.763
	1.53 (1.28, 1.83)	<0.0001	1.01 (0.79, 1.28)	0.949
Deprivation score	1.01 (1.01, 1.02)	<0.0001	1.01 (1.01, 1.02)	<0.0001
Dementia diagnostic group (AD reference)				

For peer review only - http://bmjopen.bmj.com/site/about/guidelines.xhtml

**Vascular**	1.34 (1.15, 1.56)	<0.0001	1.02 (0.84, 1.24)	0.825
**Mixed**	1.16 (0.95, 1.4)	0.145	0.8 (0.61, 1.05)	0.114
**Other**	1.12 (0.98, 1.27)	0.099	1.12 (0.98, 1.27)	0.099
**Ethnic group (European reference)**				
**Other**	1.2 (1.05, 1.37)	0.009	1.2 (1.05, 1.37)	0.009
**Pneumonia**	1.38 (1.13, 1.7)	0.002	1.38 (1.13, 1.7)	0.002
**Perforated/bleeding ulcer**	7.01 (1.56, 31.57)	0.011	7.01 (1.56, 31.57)	0.011
**Dehydration**	1.71 (0.92, 3.17)	0.09	1.71 (0.92, 3.17)	0.09
**Gastroenteritis**	0.57 (0.32, 1.02)	0.059	0.57 (0.32, 1.02)	0.059
**UTI pyelonephritis**	1.38 (1.15, 1.65)	<0.0001	1.38 (1.15, 1.65)	<0.0001

* Proportional odds assumption violated for age and dementia diagnostic group. Note that where it is not violated, the same odds ratio applies to both comparisons.

For a continuous or an ordinal variable, the odds ratio is calculated relative to a one-unit change in the variable. For ACSCs, the reference category is absence of the condition recorded.

ACSC: ambulatory care sensitive condition

Goodness of fit: Likelihood ratio test - LR Chi square 144.6, p<0.0001

Sensitivity analysis (expanded ACSC definition)

In addition to perforated/bleeding ulcer (OR 6.92), pneumonia (OR 1.38) and UTI pyelonephritis (OR 1.49), in the sensitivity analysis where the ACSC definition was relaxed, convulsion/epilepsy (OR 1.58) was also found to be significantly associated with more severe dementia (**Supplementary table 2**).

For peer review only

DISCUSSION

We evaluated all-cause and potentially avoidable-causes of hospitalisation around the times of randomly selected cognitive function assessments in people with clinically diagnosed dementia, modelling mild vs. moderate vs. severe dementia at the index dates in question. Over half of our sample had a hospitalisation of any kind during a 12-month period of evaluation, and almost a quarter had an ACSC-related hospital admission, which accounted for nearly 40% of hospitalised patients. The proportions of patients with at least one hospitalisation overall, or with one ACSC-related hospitalisation, were greater in the groups with more advanced dementia. The odds of being in a more advanced stage was almost 1.3-fold higher for those with an ACSC-related admission after adjusting for demographics, time from first dementia record at index date (a proxy for disease duration) and neighbourhood deprivation.

Around 6% of community-dwelling individuals with dementia in a US Veterans Health Administration database had at least one ACSC admission during a 12-month period; however, Medicare facility-use was not included, and hospitalisations may thus have been underestimated.[23] In another study, an average of 28% of Medicare beneficiaries per year aged ≥65 years with dementia participating in the Washington Heights-Inwood Columbia Aging

For peer review only - http://bmjopen.bmj.com/site/about/guidelines.xhtml

Project (WHICAP), had an ACSC-related admission,[8] which was more consistent with our own findings.

After adjusting for a range of clinical and demographic variables, we found that an ACSC-related admission remained associated with more advanced dementia. Hospitalisations with UTI/pyelonephritis, pneumonia and perforated/bleeding ulcer recorded as part of an admission were associated with more advanced dementia in our study. Although these have been reported as common causes of admission in people with dementia relative to those without dementia,[24-26] there is sparse literature on relationships with dementia severity. A study of Medicare beneficiaries with dementia found that that worse cognitive status was associated with higher risks of hospitalisations for diabetes, pneumonia, hypertension, and dehydration, and that worse functional status was associated with higher risks of hospitalisation for diabetes and UTI, marginally higher for dehydration, but lower for hypertension.[8]

UTI/pyelonephritis was recorded in 17.4% of hospitalised patients with dementia in our sample, and its presence was associated with more severe dementia. UTIs in more severe dementia may reflect worse immune function,[27] problems with mobility associated with urinary

For peer review only - http://bmjopen.bmj.com/site/about/guidelines.xhtml

incontinence and subsequent infection,[28] and inactivity or dehydration.[29] Consistently, we also observed a suggestion of association with dehydration in more severe dementia, although this was not significant (p = 0.09). The presence of acute urinary symptoms and laboratory evidence (such as a positive urine culture) is required to make a formal UTI diagnosis. Difficulties collecting a urine specimen, an inability to accurately report symptoms, together with the high prevalence of asymptomatic bacteriuria in people with dementia may lead to misdiagnosis, particularly in those whose dementia is severe.[30 31] Further, non-specific symptoms such as new onset or worsening of confusion is a common reason suspecting a UTI in elderly patients and may also lead to over-diagnosis in this population.[32] These factors may confound our findings and overestimate the impact of UTIs on ACSC-related admissions.

Hospitalised pneumonia was common in our sample (12.5% of hospitalised patients with dementia) and was associated with more advanced dementia, as has been described by others.[33 34] Uptake of the pneumococcal vaccine in the over-65 age-group is relatively high in the UK (69.5% of the primary care population)[35]: this suggests that pneumonia in our sample may be due to other bacterial or viral causes, or may be occurring as a result of an impaired immune system, dysphagia or urinary incontinence, which are common in, and associated with a higher

For peer review only - http://bmjopen.bmj.com/site/about/guidelines.xhtml

risk of pneumonia in, the elderly.[36 37] Furthermore, it was not possible to determine whether instances of pneumonia identified in our sample were acquired within hospital as part of the inpatient episode, or in the community prior to hospitalisation.

Perforated/bleeding ulcer related admissions were strongly associated with more severe dementia at the index date. A lack of literature on this topic, meant that we were unable to corroborate our findings. Importantly, as only 6 individuals had an admission for this ACSC, limited conclusions can be drawn from our analysis. An understanding of the use of NSAIDs (as a risk factor for GI bleeding) and use of and adherence to proton pump inhibitors which are indicated for the management and/or prevention of GI bleeding by dementia severity would be useful to provide context to our observations.

For peer review only

Lastly, we observed that the mean number of ACSC admissions amongst hospitalised patients was highest in those with moderate compared to mild or severe dementia. It is possible that this observation reflects the increased support that people with more advanced dementia receive.[38] For example, compared to those with moderate dementia, individuals with severe

For peer review only - http://bmjopen.bmj.com/site/about/guidelines.xhtml

dementia may quality for support (such as home health care) or be more likely to live in assisted accommodation or care homes where there is greater support to manage ACSCs on a day-to-day basis. For those residing in the community, caregiver burden has been reported to be associated with an increased risk of hospitalisation.[39 40] Barriers accessing care (for example individuals being too frail to be transported to hospital) and end-of-life care pathway implementation, such as individual care goals, may also influence hospitalisation patterns.

There is an existing body of evidence evaluating various models of care on the risk of hospitalisation including care management, counselling/self-help, enhanced GP services or memory clinics, and physiotherapy or occupational therapy, although none have demonstrated a clear benefit.[41] GP practice factors such as a larger practice population (which may have greater experience with dementia care and/or share patient care between GPs), quality of care, and/or an increasing proportion of the local clinical budget allotted to mental health are reported to be associated with a slight decrease in the rate of avoidable and unavoidable hospital admissions.[42] Others have reported poor continuity of care as a risk factor for hospitalisation.[43] Future care models could include a multi-agency approach including caregiver support.

Strengths and limitations

For peer review only - http://bmjopen.bmj.com/site/about/guidelines.xhtml

Our study evaluated hospitalisations in a large sample of clinically diagnosed cases of dementia where cognitive assessment is performed as part of routine clinical care. Linkage of our clinical dataset to a national hospitalisation database benefited from near-complete outcome ascertainment, since hospitalisation is free at the point of delivery in the UK context. We also benefited from information allowing differentiation between mild, moderate and severe dementia. Considering limitations, the analysed sample was from a single urban and suburban catchment and included only cases with dementia diagnosed in specialist services; however, the estimated proportion of people with dementia in the SLaM catchment who receive a specialist diagnosis is relatively high at 75.2%.[44] Considering reasons for hospitalisation, these were derived from the discharge diagnoses recorded for last episode of the hospital admission, and it was not possible to distinguish complications that arose during the hospital stay from reasons for the initial admission. Further, scrutiny of a patient's medical history prior to hospitalisation would be required to determine whether a particular ACSC-related admission was truly preventable. Thus, ACSC-related admissions serve as indicators of, rather than definitive measures of, sub-optimal care in the primary-care or community-care settings. We used an established definition of ACSC-related admissions to aid comparability with other

For peer review only

research. However, the relevance of some of these conditions for an older population is not clear. It is arguable that more specific ACSC definitions might be needed in this population.

Considering residual confounding, this analysis did not account for comorbidity burden or medication use (including polypharmacy) which are potentially associated with hospitalisations and dementia severity,[9 11 23 40 45] nor did it account for prior healthcare utilisation, functional ability,[45] or psychiatric symptoms. Presence of frailty, living arrangement, available support (family, community and home health support), care-giver burden and GP practice level factors are associated with hospitalisation [40 42] but were not available in this analysis.

Patients with severe dementia were under-represented in our sample and the time since first diagnosis in SLaM in these patients was relatively short despite the extent of cognitive impairment; it is therefore possible that our patients with severe dementia are generally those presenting late in their disease course, and not representative of the general advanced-dementia population. Our analysis evaluated associations between hospitalisation patterns and contemporaneously measured severity of dementia, so it is not appropriate to draw conclusions

For peer review only - http://bmjopen.bmj.com/site/about/guidelines.xhtml

about causation. In addition, we evaluated hospitalisation outcomes up to 6-months after a cognitive measure and did not attempt to account for survival effects. Mortality increases with severity of dementia[34], and is a competing risk for hospitalisation, although the relatively short timeframe evaluated should mitigate the effects of this.

Conclusions

Our study adds to a small but increasing body of evidence on the relationship between dementia and potentially avoidable hospitalisations. Despite the clinical and economic burden, we are not aware of any studies in the UK setting that describe potentially avoidable hospitalisations in patients with dementia. We show that potentially avoidable hospitalisations are common in people with dementia, and in particular in those with more advanced dementia; these findings are of importance given that hospitalisation is a significant element in the cost of dementia care and that the number of people with dementia is increasing. Greater attention to the extent of cognitive impairment, and not only the presence of a dementia diagnosis, is needed in order to prevent hospitalisations. However, more work is clearly needed to better understand factors contributing to the high rates of ACSC-related hospitalisation, including the management of

For peer review only

For peer review only - http://bmjopen.bmj.com/site/about/guidelines.xhtml

ACSCs in the primary care setting. Reducing admissions for ACSCs represents an opportunity to improve both the quality and efficiency of care in healthcare systems.

Ethics statements

Patient consent for publication

Not required.

Ethics approval

The Oxford Research Ethics Committee C (reference 18/SC/0372) approved CRIS at the Maudsley as a data resource for secondary analysis.

Author contributions

UG was responsible for the design, analysis, interpretation and drafting of the manuscript. GP contributed to the drafting of the manuscript. NG provided statistical input and contributed to the drafting of the manuscript. RS contributed to the design, interpretation and drafting of the manuscript

Funding

This work was supported by NIHR Biomedical Research Centre at the South London and Maudsley NHS Foundation Trust and King's College London. RS is additionally part-funded

For peer review only - http://bmjopen.bmj.com/site/about/guidelines.xhtml

by an NIHR Senior Investigator Award, by the National Institute for Health Research (NIHR) Applied Research Collaboration South London (NIHR ARC South London) at King's College Hospital NHS Foundation Trust, and by the DATAMIND HDR UK Mental Health Data Hub (MRC grant MR/W014386).

Role of the funding source

The funder of the study had no role in the study design, data collection, data analysis, data interpretation, or writing of the report. UG, GP and RS had access to the anonymised data. The corresponding author had the final responsibility for the decision to submit for publication.

Declaration of Competing Interest

UG and NG are employees of GSK, hold stock and receive a salary from GSK. RS has received research support in the last 5 years from Janssen, Takeda and GSK.

Acknowledgments

The authors would like to acknowledge Hitesh Shetty for his contributions to the study.

Data sharing

Because of their nature and to comply with their ethical approval, CRIS data are required to remain within the firewall of the South London and Maudsley NHS Foundation Trust (SLaM). Access to the data used for this study can be facilitated by the CRIS Oversight Committee on application and with appropriate SLaM affiliation, details of which can be obtained from cris.administrator@slam.nhs.uk.

For peer review only

For peer review only - http://bmjopen.bmj.com/site/about/guidelines.xhtml

References

1. Dementia Statistics Hub. Dementia Statistics. Hospitals online [Available from: https://www.dementiastatistics.org/statistics/hospitals/ accessed 19/03/2021 2021.
2. Alzheimer's Society. Analysis of NHS England's Hospital Episode Statistics dataset 2012/13 to 2017/18: Alzheimer's Society, 2020.
3. Xu JZ, Y.; Qiu, C.; Cheng, F. Global and regional economic costs of dementia: a systematic review. *The Lancet* 2017;390:S47. doi: 10.1016/S0140-6736(17)33185-9
4. Prince MK, M.; Guerchet, M.; McCrone, P.; Prina, M.; Comas-Herrera, A.; Wittenberg, R.; Adelaja, B.; Hu, B.; King, D.; Rehill, A.; Salimkumar, D.;. Dementia UK Update, 2014.
5. NHS Digital. Enhancing quality of life for people with long-term conditions. In: Team CI, ed. UK: Health and Social Care Information Centre (HSIC), 2017.
6. Bardsley M, Blunt I, Davies S, et al. Is secondary preventive care improving? Observational study of 10-year trends in emergency admissions for conditions amenable to ambulatory care. *BMJ Open* 2013;3(1) doi: 10.1136/bmjopen-2012-002007 [published Online First: 2013/01/05]
7. Blunt I. Focus on preventable admissions. Trends in emergency admissions for ambulatory care sensitive conditions, 2001 to 2013. QualityWatch Focus, 2013.
8. Zhu CW, Cosentino S, Ornstein K, et al. Use and cost of hospitalization in dementia: Longitudinal results from a community-based study. *International Journal of Geriatric Psychiatry* 2015;30(8):833-41. doi: 10.1002/gps.4222
9. Bynum JPW, Rabins PV, Weller W, et al. The Relationship between a Dementia Diagnosis, Chronic Illness, Medicare Expenditures, and Hospital Use. *Journal of the American Geriatrics Society* 2004;52(2):187-94. doi: 10.1111/j.1532-5415.2004.52054.x
10. Phelan EA, Borson S, Grothaus L, et al. Association of incident dementia with hospitalizations. *JAMA - Journal of the American Medical Association* 2012;307(2):165-72. doi: 10.1001/jama.2011.1964
11. Lin PJ, Fillit HM, Cohen JT, et al. Potentially avoidable hospitalizations among Medicare beneficiaries with Alzheimer's disease and related disorders. *Alzheimer's and Dementia* 2013;9(1):30-38. doi: 10.1016/j.jalz.2012.11.002
12. Alzheimer's Association. Reducing potentially preventable hospitalisations for people living with Alzheimer's and other dementias 2017 [Available from: https://pdfs.semanticscholar.org/9448/37faa2cb818b66264a51d4a346aa8c1a0f93.pdf.
13. Han SD, Boyle PA, James BD, et al. Mild cognitive impairment is associated with poorer decision-making in community-based older persons. *J Am Geriatr Soc* 2015;63(4):676-83. doi: 10.1111/jgs.13346 [published Online First: 2015/04/09]
14. Ibrahim JE, Anderson LJ, MacPhail A, et al. Chronic disease self-management support for persons with dementia, in a clinical setting. *J Multidiscip Healthc* 2017;10:49-58. doi: 10.2147/JMDH.S121626 [published Online First: 2017/02/10]
15. Lin PJ, Rane PB, Fillit HM, et al. National estimates of potentially avoidable hospitalizations among medicare beneficiaries with Alzheimer's disease and related dementias. *Alzheimer's and Dementia* 2016;12(7):P253-P54.
16. Perera G, Broadbent M, Callard F, et al. Cohort profile of the South London and Maudsley NHS Foundation Trust Biomedical Research Centre (SLaM BRC) Case

For peer review only - http://bmjopen.bmj.com/site/about/guidelines.xhtml

Register: current status and recent enhancement of an Electronic Mental Health Record-derived data resource. *BMJ open* 2016;6(3):e008721.
17. Jackson RG, Patel R, Jayatilleke N, et al. Natural language processing to extract symptoms of severe mental illness from clinical text: the Clinical Record Interactive Search Comprehensive Data Extraction (CRIS-CODE) project. *BMJ open* 2017;7(1):e012012.
18. NHS Digital. Hospital Episode Statistics (HES) 2019 [Available from: https://digital.nhs.uk/data-and-information/data-tools-and-services/data-services/hospital-episode-statistics accessed 13/01/2019 2019.
19. Canuto A, Weber K, Gold G, et al. Structured assessment of mental health status in psychogeriatrics: validity of the French HoNOS65+. *Can J Psychiatry* 2007;52(1):37-45. doi: 10.1177/070674370705200107 [published Online First: 2007/04/21]
20. Ministry of Housing Communities and Local Government. The English Indices of Deprivation 2019. In: Ministry of Housing CaLG, ed., 2019.
21. NICE. Donepezil, galantamine, rivastigmine and memantine for the treatment of dementia. Health technology appraisal 2018 update, 2018.
22. Williams R. Understanding and interpreting generalized ordered logit models. *The Journal of Mathematical Sociology* 2016;40:7-20.
23. Maust DT, Kim HM, Chiang C, et al. Predicting Risk of Potentially Preventable Hospitalization in Older Adults with Dementia. *Journal of the American Geriatrics Society* 2019;67(10):2077-84. doi: 10.1111/jgs.16030
24. Natalwala A, Potluri R, Uppal H, et al. Reasons for hospital admissions in dementia patients in Birmingham, UK, during 2002-2007. *Dementia and Geriatric Cognitive Disorders* 2008;26(6):499-505. doi: 10.1159/000171044
25. Rao A, Suliman A, Vuik S, et al. Outcomes of dementia: Systematic review and meta-analysis of hospital administrative database studies. *Arch Gerontol Geriatr* 2016;66:198-204. doi: 10.1016/j.archger.2016.06.008
26. Hsu CC, Hsu YC, Chang KH, et al. Association of Dementia and Peptic Ulcer Disease: A Nationwide Population-Based Study. *Am J Alzheimers Dis Other Demen* 2016;31(5):389-94. doi: 10.1177/1533317515617546 [published Online First: 2016/01/24]
27. Janssens JP. Pneumonia in the elderly (geriatric) population. *Curr Opin Pulm Med* 2005;11(3):226-30. [published Online First: 2005/04/09]
28. Schnelle JF, Leung FW. Urinary and fecal incontinence in nursing homes. *Gastroenterology* 2004;126(1 Suppl 1):S41-7. [published Online First: 2004/02/24]
29. Palmer JL, Metheny NA. Preventing aspiration in older adults with dysphagia. *Am J Nurs* 2008;108(2):40-8. doi: 10.1097/01.NAJ.0000308961.99857.33 [published Online First: 2008/01/30]
30. Ninan S, Walton C, Barlow G. Investigation of suspected urinary tract infection in older people. *BMJ : British Medical Journal* 2014;349:g4070. doi: 10.1136/bmj.g4070
31. Burkett E, Carpenter CR, Arendts G, et al. Diagnosis of urinary tract infection in older persons in the emergency department: To pee or not to pee, that is the question. *Emerg Med Australas* 2019;31(5):856-62. doi: 10.1111/1742-6723.13376 [published Online First: 2019/09/04]
32. Mayne S, Bowden A, Sundvall PD, et al. The scientific evidence for a potential link between confusion and urinary tract infection in the elderly is still confusing - a

For peer review only - http://bmjopen.bmj.com/site/about/guidelines.xhtml

systematic literature review. *BMC Geriatr* 2019;19(1):32. doi: 10.1186/s12877-019-1049-7 [published Online First: 2019/02/06]
33. Knox S, Downer B, Haas A, et al. Dementia Severity Associated With Increased Risk of Potentially Preventable Readmissions During Home Health Care. *Journal of the American Medical Directors Association* 2019 doi: 10.1016/j.jamda.2019.09.012
34. Sachs GA, Carter R, Holtz LR, et al. Cognitive impairment: an independent predictor of excess mortality: a cohort study. *Ann Intern Med* 2011;155(5):300-8. doi: 10.7326/0003-4819-155-5-201109060-00007 [published Online First: 2011/09/07]
35. NHS. Pneumococcal Polysaccharide Vaccine (PPV) coverage report, England, April 2018 to March 2019. In: England PH, ed., 2019.
36. Marrie TJ. Community-acquired pneumonia in the elderly. *Clinical Infectious Diseases* 2000;31(4):1066-78. doi: 10.1086/318124
37. Chong CP, Street PR. Pneumonia in the elderly: a review of the epidemiology, pathogenesis, microbiology, and clinical features. *South Med J* 2008;101(11):1141-5; quiz 32, 79. doi: 10.1097/SMJ.0b013e318181d5b5 [published Online First: 2008/12/18]
38. Fillenbaum G, Heyman A, Peterson BL, et al. Use and cost of hospitalization of patients with AD by stage and living arrangement: CERAD XXI. *Neurology* 2001;56(2):201-6. [published Online First: 2001/02/13]
39. Voisin T, Andrieu S, Cantet C, et al. Predictive factors of hospitalizations in Alzheimer's disease: A two-year prospective study in 686 patients of the REAL.FR study. *Journal of Nutrition, Health and Aging* 2010;14(4):288-91. doi: 10.1007/s12603-010-0063-4
40. Afonso-Argilés FJ, Meyer G, Stephan A, et al. Emergency department and hospital admissions among people with dementia living at home or in nursing homes: results of the European RightTimePlaceCare project on their frequency, associated factors and costs. *BMC Geriatr* 2020;20(1):453. doi: 10.1186/s12877-020-01835-x [published Online First: 2020/11/07]
41. Packer R, Ben Shlomo Y, Whiting P. Can non-pharmacological interventions reduce hospital admissions in people with dementia? A systematic review. *PLOS ONE* 2019;14(10):e0223717. doi: 10.1371/journal.pone.0223717
42. Eyles E, Redaniel MT, Purdy S, et al. Associations of GP practice characteristics with the rate of ambulatory care sensitive conditions in people living with dementia in England: an ecological analysis of routine data. *BMC Health Serv Res* 2021;21(1):613. doi: 10.1186/s12913-021-06634-7 [published Online First: 2021/06/30]
43. Amjad H, Carmichael D, Austin AM, et al. Continuity of care and health care utilization in older adults with dementia in fee-for-service medicare. *JAMA Internal Medicine* 2016;176(9):1371-78. doi: 10.1001/jamainternmed.2016.3553
44. NHS England. Dementia diagnosis monthly workbook: Secondary dementia diagnosis monthly workbook 2017, 2017.
45. Shepherd H, Livingston G, Chan J, et al. Hospitalisation rates and predictors in people with dementia: A systematic review and meta-analysis. *BMC Medicine* 2019;17(1) doi: 10.1186/s12916-019-1369-7

For peer review only

Supplementary Table 1: Ambulatory care sensitive conditions*

ACSC	ICD code position		ICD-10 codes
	Main analysis	Sensitivity analysis	
**Angina**	Principal	1° or 2°	I20, I200, I201, I208, I209, I240, I248, I249, I25, I250, I251, I252, I253, I254, I255, I256, I258, I259
**Asthma**	Principal	1° or 2°	J45, J450, J451, J458, J459, J46, J46X
**Cellulitis**	Principal	1° or 2°	L03, L030, L031, L032, L033, L038, L039, L04, L040, L041, L042, L043, L048, L049, L08, L080, L081, L088, L089, L88, L88X, L980, L983
**Congestive heart failure**	Principal	1° or 2°	I110, I13, I130, I131, I132, I139, I50, I500, I501, I509, J81, J81X
**Convulsions and epilepsy**	Principal	1° or 2°	G40, G400, G401, G402, G403, G404, G405, G406, G407, G408, G409, G41, G410, G411, G412, G418, G419, O15, R56, R560, R568
**COPD**	Principal	1° or 2°	J20, J41, J410, J411, J418, J42, J42X, J43, J430, J431, J432, J438, J439, J44, J440, J441, J448, J449, J47, J47X
**Dehydration**	Principal	1° or 2°	E86, E86X
**Dental conditions**	Principal	1° or 2°	A690, K02, K020, K021, K022, K023, K024, K028, K029, K03, K030, K031, K032, K033, K034, K035, K036, K037, K038, K039, K04, K040, K041, K042, K043, K044, K045, K046, K047, K048, K049, K05, K050, K051, K052, K053, K054, K055, K056, K06, K060, K061, K062, K068, K069, K08, K080, K081, K082, K083, K088, K089, K09, K090, K091, K092, K098, K099, K12, K120, K121, K122, K13, K130, K131, K132, K133, K134, K135, K136, K137
**Diabetes complications**	Any	Any	E10, E100, E101, E102, E103, E104, E105, E106, E107, E108, E11, E110, E111, E112, E113, E114, E115, E116, E117, E118, E12, E120, E121, E122, E123, E124, E125, E126, E127, E128, E13, E130, E131, E132, E133, E134, E135, E136, E137, E138, E14, E140, E141, E142, E143, E144, E145, E146, E147, E148
**Ear, nose and throat infections**	Principal	1° or 2°	H66, H660, H661, H662, H663, H664, H669, H67, H670, H671, H678, J02, J020, J028, J029, J03, J030, J038, J039, J06, J060, J068, J069, J312
**Gangrene**	Any	Any	R02
**Gastroenteritis**	Principal	1° or 2°	K522, K528, K529
**Hypertension**	Principal	1° or 2°	I10, I10X, I119, I129, I139
**Influenza**	Any	Any	J10, J100, J101, J108, J11, J110, J111, J118
**Iron deficiency anaemia**	Principal	1° or 2°	D50, D501, D508, D509
**Nutritional deficiencies**	Principal	1° or 2°	E40, E40X, E41, E41X, E42, E42X, E43, E43X, E55, E550, E559, E64, E640, E641, E642, E643, E648, E649
**Nutritional deficiencies OR other nutritional deficiency anaemia**	Principal	1° or 2°	D51, D510, D511, D512, D513, D518, D519
**Other vaccine preventable**	Any	Any	A35, A35X, A36, A360, A361, A362, A363, A368, A369, A37, A370, A390, A80, A801, A802, A803, A804, A809, B05, B050, B051, B052, B053, B054, B058, B059, B06, B060, B068, B069, B16, B160, B161, B162, B169, B180, B181, B26, B260, B261, B262, B263, B268, B269, G000, G001, M010, M014
**Pelvic inflammatory disease**	Principal	1° or 2°	N70, N700, N701, N709, N73, N730, N731, N732, N733, N734, N735, N736, N738, N739, N74, N740, N741, N742, N743, N744, N748
**Perforated/bleeding ulcer**	Principal	1° or 2°	K25, K250, K251, K252, K254, K255, K256, K26, K260, K261, K262, K264, K265, K266, K27, K270, K271, K272, K274, K275, K276, K28, K280, K281, K282, K284, K286
**Pneumonia**	Any	Any	J13, J14, J153, J154, J157, J159, J168, J18, J180, J181, J182, J188, J189
**Tuberculosis**	Principal	1° or 2°	A15, A150, A151, A152, A153, A154, A155, A156, A157, A158, A159, A16, A160, A161, A162, A163, A164, A165, A167, A168, A169, A19, A190, A191, A192, A198, A199
**Urinary tract infection /Pyelonephritis**	Principal	1° or 2°	N10, N10X, N11, N110, N111, N118, N119, N12, N12X, N136, N30, N300, N301, N302, N303, N304, N308, N309, N390

ACSC: ambulatory care sensitive condition

*This table was created by the authors, however, the ACSCs, the respective ICD-10 codes and their position were initially based on those published by Bardsley *et al*, 2013. Codes were augmented with those published by the UK Health and Social Care Information Centre and by the authors. Deviations from the approach of Bardsley *et al* comprised of: i) cystitis codes were added to the UTI category; ii) O15 (eclampsia) was removed from the convulsions and epilepsy category. The approach used in the sensitivity was determined by the authors.

For peer review only

Supplementary Table 2: Results from stepwise-selected generalised ordinal logistic regression model for severity of dementia (sensitivity analysis *)

	Moderate/severe vs. mild dementia		Severe vs. mild/moderate dementia	
	**OR (95% CI)**	**p**	**OR (95% CI)**	**p**
**Sex (male reference)**	1.09 (0.97, 1.21)	0.141	1.09 (0.97, 1.21)	0.141
**Time since first presentation at SLaM**	1.13 (1.08, 1.18)	<0.001	1.13 (1.08, 1.18)	<0.001
**Age group at index date**	1.21 (1.11, 1.31)	<0.001	0.99 (0.89, 1.11)	0.856
**Ethnic group (European reference)**				
	1.21 (1.06, 1.38)	0.005	1.21 (1.06, 1.38)	0.005
**Dementia diagnostic group (AD reference)**				
Vascular	1.33 (1.15, 1.56)	<0.001	1.02 (0.85, 1.24)	0.816
Mixed	1.15 (0.95, 1.4)	0.163	0.8 (0.61, 1.05)	0.107
Other	1.11 (0.98, 1.27)	0.112	1.11 (0.98, 1.27)	0.112
**Deprivation score**	1.12 (1.06, 1.17)	<0.001	1.12 (1.06, 1.17)	<0.001
**Convulsions**	1.58 (0.96, 2.58)	0.07	1.58 (0.96, 2.58)	0.07
**Iron deficiency anaemia**	1.23 (0.63, 2.4)	0.542	0.25 (0.06, 1.05)	0.058
**Perforated/bleeding ulcer**	6.92 (1.56, 30.77)	0.011	6.92 (1.56, 30.77)	0.011
**Pneumonia**	1.38 (1.12, 1.69)	0.002	1.38 (1.12, 1.69)	0.002
**UTI pyelonephritis**	1.29 (1.1, 1.51)	0.002	1.29 (1.1, 1.51)	0.002

Goodness of fit: LR Chi square 143.3, $p<0.0001$
ACSC: ambulatory care sensitive condition
*Criteria were relaxed to include diagnoses in either of the first two positions for ACSCs that were initially defined based on a diagnosis in the primary position only.

For peer review only

STROBE 2007 (v4) checklist of items to be included in reports of observational studies in epidemiology*
Checklist for cohort, case-control, and cross-sectional studies (combined)

Section/Topic	Item #	Recommendation	Reported on page #
**Title and abstract**	1	(*a*) Indicate the study's design with a commonly used term in the title or the abstract	1
		(*b*) Provide in the abstract an informative and balanced summary of what was done and what was found	2
**Introduction**			
Background/rationale	2	Explain the scientific background and rationale for the investigation being reported	5
Objectives	3	State specific objectives, including any pre-specified hypotheses	5
**Methods**			
Study design	4	Present key elements of study design early in the paper	6
Setting	5	Describe the setting, locations, and relevant dates, including periods of recruitment, exposure, follow-up, and data collection	6
Participants	6	(*a*) *Cohort study*—Give the eligibility criteria, and the sources and methods of selection of participants. Describe methods of follow-up<br>*Case-control study*—Give the eligibility criteria, and the sources and methods of case ascertainment and control selection. Give the rationale for the choice of cases and controls<br>*Cross-sectional study*—Give the eligibility criteria, and the sources and methods of selection of participants	6
		(*b*) *Cohort study*—For matched studies, give matching criteria and number of exposed and unexposed<br>*Case-control study*—For matched studies, give matching criteria and the number of controls per case	
Variables	7	Clearly define all outcomes, exposures, predictors, potential confounders, and effect modifiers. Give diagnostic criteria, if applicable	8-9
Data sources/ measurement	8*	For each variable of interest, give sources of data and details of methods of assessment (measurement). Describe comparability of assessment methods if there is more than one group	*8-9*
Bias	9	Describe any efforts to address potential sources of bias	
Study size	10	Explain how the study size was arrived at	N/A
Quantitative variables	11	Explain how quantitative variables were handled in the analyses. If applicable, describe which groupings were chosen and why	8-9
Statistical methods	12	(*a*) Describe all statistical methods, including those used to control for confounding	10-11
		(*b*) Describe any methods used to examine subgroups and interactions	10-11
		(*c*) Explain how missing data were addressed	N/A
		(*d*) *Cohort study*—If applicable, explain how loss to follow-up was addressed<br>*Case-control study*—If applicable, explain how matching of cases and controls was addressed	

		Cross-sectional study—If applicable, describe analytical methods taking account of sampling strategy	
		(*e*) Describe any sensitivity analyses	9
**Results**			
Participants	13*	(a) Report numbers of individuals at each stage of study—eg numbers potentially eligible, examined for eligibility, confirmed eligible, included in the study, completing follow-up, and analysed	
		(b) Give reasons for non-participation at each stage	
		(c) Consider use of a flow diagram	
Descriptive data	14*	(a) Give characteristics of study participants (eg demographic, clinical, social) and information on exposures and potential confounders	12
		(b) Indicate number of participants with missing data for each variable of interest	N/A
		(c) *Cohort study*—Summarise follow-up time (eg, average and total amount)	
Outcome data	15*	*Cohort study*—Report numbers of outcome events or summary measures over time	15
		Case-control study—Report numbers in each exposure category, or summary measures of exposure	
		Cross-sectional study—Report numbers of outcome events or summary measures	
Main results	16	(*a*) Give unadjusted estimates and, if applicable, confounder-adjusted estimates and their precision (eg, 95% confidence interval). Make clear which confounders were adjusted for and why they were included	16
		(*b*) Report category boundaries when continuous variables were categorized	
		(*c*) If relevant, consider translating estimates of relative risk into absolute risk for a meaningful time period	
Other analyses	17	Report other analyses done—eg analyses of subgroups and interactions, and sensitivity analyses	
**Discussion**			
Key results	18	Summarise key results with reference to study objectives	19
Limitations	19	Discuss limitations of the study, taking into account sources of potential bias or imprecision. Discuss both direction and magnitude of any potential bias	21
Interpretation	20	Give a cautious overall interpretation of results considering objectives, limitations, multiplicity of analyses, results from similar studies, and other relevant evidence	22
Generalisability	21	Discuss the generalisability (external validity) of the study results	21
**Other information**			
Funding	22	Give the source of funding and the role of the funders for the present study and, if applicable, for the original study on which the present article is based	23

*Give information separately for cases and controls in case-control studies and, if applicable, for exposed and unexposed groups in cohort and cross-sectional studies.
Note: An Explanation and Elaboration article discusses each checklist item and gives methodological background and published examples of transparent reporting. The STROBE checklist is best used in conjunction with this article (freely available on the Web sites of PLoS Medicine at http://www.plosmedicine.org/, Annals of Internal Medicine at http://www.annals.org/, and Epidemiology at http://www.epidem.com/). Information on the STROBE Initiative is available at www.strobe-statement.org.

For peer review only
